# Association of cyclin-dependent kinase inhibitor 2B antisense RNA 1 gene expression and rs2383207 variant with breast cancer risk and survival

**DOI:** 10.1186/s11658-021-00258-9

**Published:** 2021-04-13

**Authors:** Shahad W. Kattan, Yahya H. Hobani, Sameerah Shaheen, Sara H. Mokhtar, Mohammad H. Hussein, Eman A. Toraih, Manal S. Fawzy, Hussein Abdelaziz Abdalla

**Affiliations:** 1 Department of Medical Laboratory, College of Applied Medical Sciences, Taibah University, Yanbu, Saudi Arabia; 2 Department of Medical Laboratory Technology, College of Applied Medical Sciences, Jazan University, Jazan, Kingdom of Saudi Arabia; 3 Anatomy Department and Stem Cell Unit, College of Medicine, King Saud University, Riyadh, Saudi Arabia; 4 Department of Medical Laboratory Technology, Faculty of Applied Medical Sciences, King Abdulaziz University, Jeddah, Saudi Arabia; 5 Department of Surgery, Tulane University, School of Medicine, New Orleans, LA USA; 6 Genetics Unit, Department of Histology and Cell Biology, Faculty of Medicine, Suez Canal University, Ismailia, Egypt; 7 Department of Medical Biochemistry and Molecular Biology, Faculty of Medicine, Suez Canal University, Ismailia, Egypt; 8 Department of Biochemistry, College of Medicine, Northern Border University, Arar, Saudi Arabia; 9 Department of Medical Biochemistry, Faculty of Medicine, Taibah University, Medina, Saudi Arabia; 10 Department of Medical Biochemistry, Faculty of Medicine, Mansoura University, Mansoura, Egypt

**Keywords:** Breast cancer, CDKN2B-AS1, Gene expression, Long non-coding RNA, Single nucleotide polymorphism, Survival

## Abstract

**Background:**

The expression signature of deregulated long non-coding RNAs (lncRNAs) and related genetic variants is implicated in every stage of tumorigenesis, progression, and recurrence. This study aimed to explore the association of lncRNA cyclin-dependent kinase inhibitor 2B antisense RNA 1 (*CDKN2B-AS1*) gene expression and the rs2383207A>G intronic variant with breast cancer (BC) risk and prognosis and to verify the molecular role and networks of this lncRNA in BC by bioinformatics gene analysis.

**Methods:**

Serum CDKN2B-AS1 relative expression and rs2383207 genotypes were determined in 214 unrelated women (104 primary BC and 110 controls) using real-time PCR. Sixteen BC studies from The Cancer Genome Atlas (TCGA) including 8925 patients were also retrieved for validation of results.

**Results:**

CDKN2B-AS1 serum levels were upregulated in the BC patients relative to controls. A/A genotype carriers were three times more likely to develop BC under homozygous (OR = 3.27, 95% CI 1.20–8.88, *P* = 0.044) and recessive (OR = 3.17, 95% CI 1.20–8.34, *P* = 0.013) models. G/G homozygous patients had a higher expression level [median and quartile values were 3.14 (1.52–4.25)] than A/G [1.42 (0.93–2.35)] and A/A [1.62 (1.33–2.51)] cohorts (*P* = 0.006). The Kaplan–Meier curve also revealed a higher mean survival duration of G/G cohorts (20.6 months) compared to their counterparts (A/A: 15.8 and A/G: 17.2 months) (*P* < 0.001). Consistently, BC data sets revealed better survival in cohorts with high expression levels (*P* = 0.003). Principal component analysis (PCA) showed a deviation of patients who had shorter survival towards A/A and A/G genotypes, multiple lesions, advanced stage, lymphovascular invasion, and HER2^+^ receptor staining. Ingenuity Pathway Analysis (IPA) showed key genes highly enriched in BC with *CDKN2B-AS1*.

**Conclusions:**

The findings support the putative role of CDKN2B-AS1 as an epigenetic marker in BC and open a new avenue for its potential use as a therapeutic molecular target in this type of cancer.

**Supplementary Information:**

The online version contains supplementary material available at 10.1186/s11658-021-00258-9.

## Introduction

According to recent cancer statistics, an estimated one-third of newly diagnosed female cancers will be breast cancers (BC) in 2020 [1]. Although the progress in diagnosis, surgical techniques, and targeted therapy has improved BC prognosis, it still represents one of the leading causes of cancer death among females aged 20–59 years [1, 2]. Hence, it is essential to explore the role of the newly emerged (epi)genetic molecular players in the BC scenario to improve disease prognosis and survival [3].

Extensive mammalian genomic and transcriptomic analyses have revealed a cluster of long non-coding RNAs (lncRNAs; > 200 nucleotides in length), which are implicated in several genetic and epigenetic regulations of the cells [4–6]. This family of non-coding RNAs can be classified into five classes: sense, antisense, bidirectional, intronic, and intergenic lncRNAs [7]. A deregulated lncRNA expression signature was found to be implied in every stage of BC tumorigenesis, progression, and recurrence [8] (Additional file 1: Table S1). Given the stability of serum/plasma lncRNAs and their RNase resistance, many studies have explored their potential use as putative epigenetic markers for cancer diagnosis/prognosis, with promising results [9–11].

**Fig. 1:**
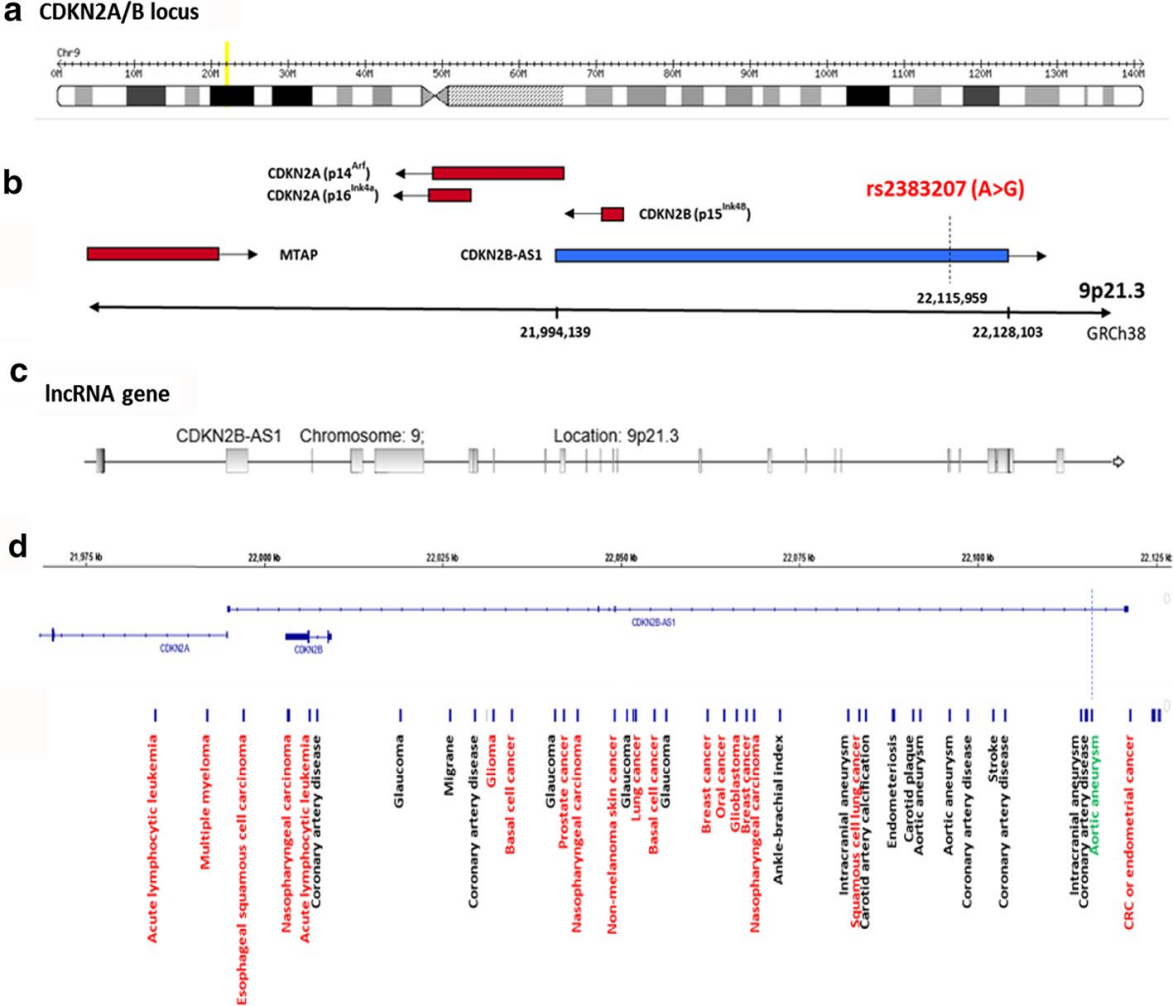
Genomic structure of *CDKN2B-AS1* locus and the common genetic single nucleotide polymorphisms with phenotypic traits. **a** *CDKN2B-AS1* is located at the human *CDKN2A/B* locus at 9p21.3, and spans a nearly 350 kb genomic region. **b** It contains three protein-coding genes (red) along with the *CDKN2B-AS1* lncRNA (blue). The protein-coding genes include (1) *S*-methyl-5′-thioadenosine phosphorylase (*MTAP*), (2) *CDKN2A* that encodes splice variants p16^INK4A^ and p14^ARF^", and (3) *CDKN2B* that encodes p15^INK4B^". *MTAP* lies at one end of the locus, 192 kb telomeric to the 5′ start of *CDKN2B-AS1*. The selected variant for the present study is shown. *ARF* alternate reading frame, *CDK* cyclin-dependent kinase. **c** At the centromeric end of the locus, *CDKN2B-AS1* has a 19–21 exon span over a 126 kb region. *CDKN2A* lies between *CDKN2B-*AS1and MTAP. The *CDKN2B-AS1* first exon is located 300 bp upstream of ARF in the transcription start site; *CDKN2B* (two exons) is located within the *CDKN2B-AS1* first intron, in an antisense direction. (Data source: http://ensembl.org). **d** The common genetic single nucleotide polymorphisms with phenotypic traits from Genome-Wide Association Studies screening for 9p21.3 locus. Diseases (black) and cancer (red) traits are illustrated. The studied SNP (green) is shown near the end of the CDKN2B-AS1 gene (Data source: http://igv.org/)

The lncRNA cyclin-dependent kinase inhibitor 2B antisense RNA 1; CDKN2B-AS1, NONCODE Gene ID: NONHSAG051899.2, also known as ANRIL (antisense non-coding RNA in the INK4 locus) was identified in a genomic hotspot region at chromosome 9p21.3 (Fig. 1a). This region is well known to be associated with human cancers [12] and was chosen based on screening of the previous studies and our detailed in silico analysis. The gene includes 19–21 exons spanning around 134 kb in the genome (Fig. 1b, c), and the coded lncRNA was reported to interact with the polycomb repressive complex-1 (PRC1) and -2 (PRC2) proteins in invasive BC [13]. These latter protein complexes play essential roles in cell differentiation and carcinogenesis [14].

CDKN2B-AS1 deregulation has been implicated in several pathological processes, including increased cellular proliferation, deregulated metabolic activity, inflammation, and decreased apoptosis [15–17]. Silencing this lncRNA was reported to prevent fibroblast and smooth muscle cell proliferation [15, 16]. Furthermore, increased CDKN2B-AS1 levels in several cancers, including BC tissues, suggest its putative role in promoting tumorigenesis [15, 18–20].

Accumulating evidence suggests that lncRNAs gene polymorphisms are also associated with cancer risk [21] and can influence lncRNA gene expression and/or function [22]. Additionally, studies have revealed that lncRNA variants may impact mRNAs splicing and stability, with subsequent changes in their cellular behavior and interacting partners [21, 23].

Given the association of *CDKN2B-AS1* expression and variants with cancer and the limitation of related studies in our region, the current study aimed to (1) investigate the impact of circulating *CDKN2B-AS1* expression and the intronic variant rs2383207 A>G (which has not been studied before) on BC risk and prognosis, (2) execute bioinformatics analysis for the specified lncRNA and (3) verify the present results against data in The Cancer Genomic Atlas (TCGA).

## Subjects and methods

### Study population

The institutional research ethics committee of the Faculty of Medicine, Suez Canal University, Ismailia, Egypt, approved the current work (Approval no. 3960). Before the start of the study, written informed consent was obtained from all participants. A total of 214 unrelated women (110 consecutive primary BC and 104 controls) were included in this study. The patients were recruited from the General Surgery Department and Oncology Diagnostic Unit, Suez Canal University Hospitals, Ismailia, Egypt. They were diagnosed clinically, radiologically, and confirmed by biopsy [24]. The patients did not have a history of receiving any treatments such as chemotherapy, radiotherapy, hormonal therapy, or immunotherapy before blood sampling. Patients with other malignancies or chronic diseases were excluded. Healthy blood donors without any signs of chronic diseases, recent pregnancy, or lactation in the last 2 years and/or associated malignancy were included as controls.

### Histopathological and immunohistochemical assessment

Specimens from BC tissue were histopathologically analyzed after the operations. Assessment of pathological grade and clinical stage were performed using the Elston and Ellis modification of the Scarff–Bloom–Richardson system and the American Joint Committee on Cancer (AJCC) tumor-lymph node-metastasis (TNM) staging system [25]. Assessment of hormonal receptors for molecular subtyping was investigated [26]. Estrogen receptor (ER) and progesterone receptor (PR) evident nuclear staining in ≥ 10% of the tumor cells was reported as positive (+ve), and if absent or < 10% staining was recorded as negative (−ve) [27]. At the same time, human epidermal growth factor receptor 2 (HER2)/neu expression was semi-quantified by the following membrane scoring system: 0, no staining or membrane staining in < 10% of tumor cells; 1+, faint or partly stained membranes in ≥ 10% of tumor cells; 2+, weak to moderate complete membrane staining in ≥ 10% of tumor cells; 3+, strong complete membrane staining in ≥ 10% of tumor cells; and accordingly, samples were classified into different molecular subtypes [22]. Then patients were subdivided into four molecular subgroups: luminal A, luminal B, human epidermal growth factor receptor 2 (HER2^+^), and triple-negative BC, as previously detailed [28, 29].

### Clinical assessment and prognostic evaluation

Clinical features, risk factor assessment, and investigations were evaluated. The Nottingham Prognostic Index (NPI) and the Immunohistochemical Prognostic Index (IHPI) were applied as previously described [28], and patients were classified accordingly to have a good, moderate, and poor prognosis. The follow-up period was extended for up to 3 years for overall survival (OS) and disease-free survival (DFS) assessment.

### Sample collection

Venous blood (5 ml) samples were collected into plain and ethylenediamine tetra-acetic acid (EDTA) Vacutainers. The samples were preserved after collection for 30 min to 2 h in the refrigerator to allow blood clotting. After that, all samples were centrifuged for 10 min. The obtained serum from plain tubes was divided into aliquots and stored at − 80 °C. One freeze–thaw cycle was carried out for the samples. The EDTA tubes were used for genomic DNA analysis.

### Nucleic acids extraction

From the serum samples, total RNAs were isolated via the Qiagen miRNeasy Serum/Plasma Kit (Qiagen, Clinilab Co., Catalog no. 217184), and from the buffy coat of EDTA blood samples, the genomic DNA was isolated via the QIAamp DNA Blood Mini kit (Catalog No. 51104; Qiagen) following the instructions of the vendors. The purity/concentrations of nucleic acids were assessed using the NanoDrop ND-1000 spectrophotometer (NanoDrop Tech., Inc. Wilmington, DE, USA). Furthermore, the integrity of the nucleic acids was tested by agarose gel electrophoresis.

### Reverse-transcription and quantitative polymerase chain reaction analysis

The Minimum Information for Publication of Quantitative Real-Time PCR Experiments (MIQE) guideline was followed during the quantitative real-time reverse-transcription polymerase chain reaction (qRT-PCR) runs [30]. The first step of the amplification process was the reverse transcription (RT) of 1 µg of total RNA to yield complementary DNA (cDNA) using a High-Capacity cDNA RT Kit (P/N 4368814, Applied Biosystems, Foster City, California, USA). The second step is the real-time PCR reaction using a specific TaqMan probe for the lncRNA CDKN2B-AS1 (Assay no. Hs04406279_m1), compared to the housekeeping gene glyceraldehyde 3-phosphate dehydrogenase (*GAPDH*) for normalization of the data. In each run, appropriate negative controls were applied: no template and no reverse transcriptase controls. The final volume of the reaction (20 µl) contained RT products (1.33 µl), 2× TaqMan Universal PCR Master Mix (10 µl), and TaqMan RNA assay (1 µl). The StepOne Real-Time PCR machine (Applied Biosystems) was set up as follows: 10 min at 95 °C, followed by 40 cycles of 15 s at 92 °C and 60 s at 60 °C [31].

### *CDKN2B-AS1* expression data analysis

The quantification cycle (C_q_) is the cycle number at which the fluorescence passed a fixed threshold [30]. The relative amount of the study lncRNA to *GAPDH* in patients compared to controls was calculated using the formula: 2^−ΔΔCq^; where ΔΔC_q_ = (C_q_ *CDKN2B-AS1* − C_q_ *GAPDH*)_BC_ − (C_q_ *CDKN2B-AS1* − C_q_ *GAPDH*)_mean controls_ [32].

### Allelic discrimination analysis

TaqMan Genotyping PCR Master Mix, No. UNG (4440043), and TaqMan single nucleotide polymorphism (SNP) Genotyping Assay Mix (assay IDC__15789010_20, Catalog# 4351379, Thermo Fisher Scientific) were used for the rs2383207 real-time allele discrimination assay. Briefly, the extracted DNA (20 ng) was diluted to 11.25 μl with DNase-free water, then added to the reaction mix containing TaqMan Master mix (12.5 μl) and TaqMan SNP Genotyping Assay (20×) Mix (1.25 µl). The PCR program was described previously [33]. The investigators were not aware of the sample status (patient versus control) during the genotyping. In each run, non-template and TaqMan enzyme negative controls were included. Ten percent of samples were tested in duplicate with a 100% concordance rate for genotype calls. The SDS software version 1.3.1 (Applied Biosystems) was applied for genotype calling.

### Genomic and functional analysis of *CDKN2B-AS1* gene

Gene locus analysis was performed in the Ensembl Genome Browser (http://ensemble.org), a database that annotates genes, transcripts, and genomic variations, computes multiple alignments, predicts regulatory function, and collects disease-related data. Variant analysis of *CDKN2B-AS1* and the impact of each were determined in the Varsome web application (https://varsome.com/), a search engine, aggregator, and impact analysis tool for human genetic variation. It displays a detailed annotation of the queried variant, including multiple notations, predicted pathogenicity status from various tools, and genomic context. Genetic variants of the *CDKN2B-AS1* gene with known pathogenicity were confirmed in ClinVar and PubMed. The predicted and tested *CDKN2B-AS1*-related SNPs’ impact on gene expression was determined from the literature and the LDHap project, a haplotype map project of the human genome that describes the common genetic patterns’ variation associated with human disease. Ensembl.org and varsome.com were used to analyze the structural and functional impact of the selected rs2383207 intron variant. The Integrative Genomics Viewer (http://igv.org/) web application was run to view the locations and Genome-Wide Association Studies (GWAS)-associated phenotypic traits in the *CDKN2A/B* locus.

The Gene-Tissue Expression (GTEx) Portal (http://gtexportal.org) and BioGPS database (http://biogps.org) were used to identify the gene expression pattern of the gene across diverse normal human tissues from the U133plus2 Affymetrix microarray experiments. The expression level of CDKN2B-AS1 in BC tissues with different molecular subtypes was determined from Expression Atlas (www.ebi.ac.uk/), an open resource that assists in finding information about gene and protein expression. Localization of the lncRNA was detected in the Compartment subcellular localization database (www.compartments.jensenlab.org/).

To retrieve high-throughput experiments in public repositories, 16 BC studies (total number of patients = 8925) in The Cancer Genomic Atlas (TCGA) were downloaded from the cBioPortal for Cancer Genomics database (http://cBioportal.org) to identify the rate of genetic alterations in the *CDKN2B-AS1* genome and aberrant expression of the gene in BC. The online Kaplan–Meier plotter program (http://kmplot.com) was utilized to plot Kaplan–Meier survival curves for *CDKN-AS1* expression from the datasets stored in this database [34]. Next, to identify the predictive role of the *CDKN2B-AS1* gene in the therapeutic response, a receiver operator characteristics (ROC) plotter tool (http://rocplot.org/) was used to link gene expression and response to therapy using transcriptome-level data of 3104 BC patients [35]. Lnc2Cancer version 2.0, a manually curated database, was used to identify experimentally supported associations between lncRNA and human cancer. It provides information on circulating, drug-resistant, prognostic lncRNAs in cancer [36].

The Gene Ontology Annotation (GOA) database was used to define the gene ontology terms related to the *CDKN2B-AS1* gene. Next, the Automatic Cancer Hallmarks Analytics Tool (CHAT) was used for classification of the PubMed literature according to the cancer taxonomy hallmarks. Breast cancer and CDKN2B-AS1 (data are shown as NPMI; normalized pointwise mutual information) were compared. The LncMAP lncRNA Modulator Atlas in Pan-cancer (http://bio-bigdata.hrbmu.edu.cn/LncMAP/index.jsp) was used to determine putative transcription factors (TFs). NetworkAnalyst (www.networkanalyst.ca) version 3.0 was used to construct gene regulatory networks (GRN) for the upstream regulators of the *CDKN2B-AS1* gene. The TF targets derived from the JASPAR TF binding site profile database and inferred from integrating the literature curated Chip-X database were identified. Also, the literature curated regulatory interaction information was collected from the RegNetwork repository [37]. IPA was employed to identify the most relevant spatial molecular interactions within the cell, predict the direction of the downstream effect in BC, and the activation and inhibition of upstream TFs. Finally, ENCORI for the RNA interactome (http://starbase.sysu.edu.cn/) with default settings presented the ceRNA (competing endogenous RNA) networks from multiple interactions of miRNA targets in pan-cancer supported by CLIP-seq data. The results were examined at a *P*-value ≤ 0.01 and the false discovery rate (FDR) ≤ 0.01 [38].

### Statistical analysis

When G power-3 software (http://www.gpower.hhu.de/) was used for study power estimation with a medium effect size, specified study design, and allowable error rate (alpha error = 0.05), it yielded 95% study power. Data were expressed as number and percentage or mean ± standard deviation for categorical and continuous data. Data were checked for normality, outliers, and skewness and were transformed in case of violation of the normality assumption. Appropriate non-parameter tests were then applied and presented as median and quartiles. Two-sided chi-square, Fisher's exact, Student t-test, one-way ANOVA, Mann–Whitney U, or Kruskal–Wallis tests were used. Construction of contingency tables was performed for genotype distribution and allelic frequency comparison. The Hardy–Weinberg equilibrium (HWE) was applied using the χ^2^ test to assess genotype distribution in all study subjects. Six genetic association models were analyzed as previously described [39]. Odds ratios (ORs) with 95% confidence intervals (CIs) and corresponding *P*-values were computed by logistic regression analysis and adjusted by the patients' demographic, clinical, and pathological characteristics. The statistical significance was set at *P*-value < 0.05. All statistical analyses were executed using SPSS version 26.0 and GraphPad Prism version 8.0. SNPStats (https://www.snpstats.net/start.htm) was applied for genetic analysis. Kaplan–Meier curves were plotted for *CDKN-AS1* genotypes with overall survival. In RStudio, correlation plots were constructed using the corrplot package, while principal component analysis (PCA) was performed using the factoextr and FactoMineR packages.

## Results

### Baseline characteristics of the study participants

The mean age of BC women was 44.3 ± 12.2 years. Only one-third (30.9%) had a positive family history of cancer. Two-thirds (63.6%) were overweight/obese. Most of the patients had a sedentary lifestyle (90%); nevertheless, only 11.8% had night shifts at work, and 7.3% of women had a prior history of breast problems. About 64.5% had early onset of menarche. A few of them had late first gravida (4.5%), nullipara (12.7%), and breastfed (16.4%), while one-third of patients were at the post-menopausal state at the time of diagnosis.

### *CDKN2B-AS1* expression profile

Based on screening 52 cases (who had available extracted RNA matched with the DNA samples for further genotype-expression correlation analysis) and 104 controls, circulatory CDKN2B-AS1 was upregulated in cancer patients compared to controls (P < 0.001). The median log fold change of CDKN2B-AS1 in the BC cohort was 1.82 (IQR: 1.38–3.71) relative to the controls.

### Allelic discrimination analysis

#### Allele and genotype frequencies of CDKN2B-AS1(rs2383207) variant

**Fig. 2:**
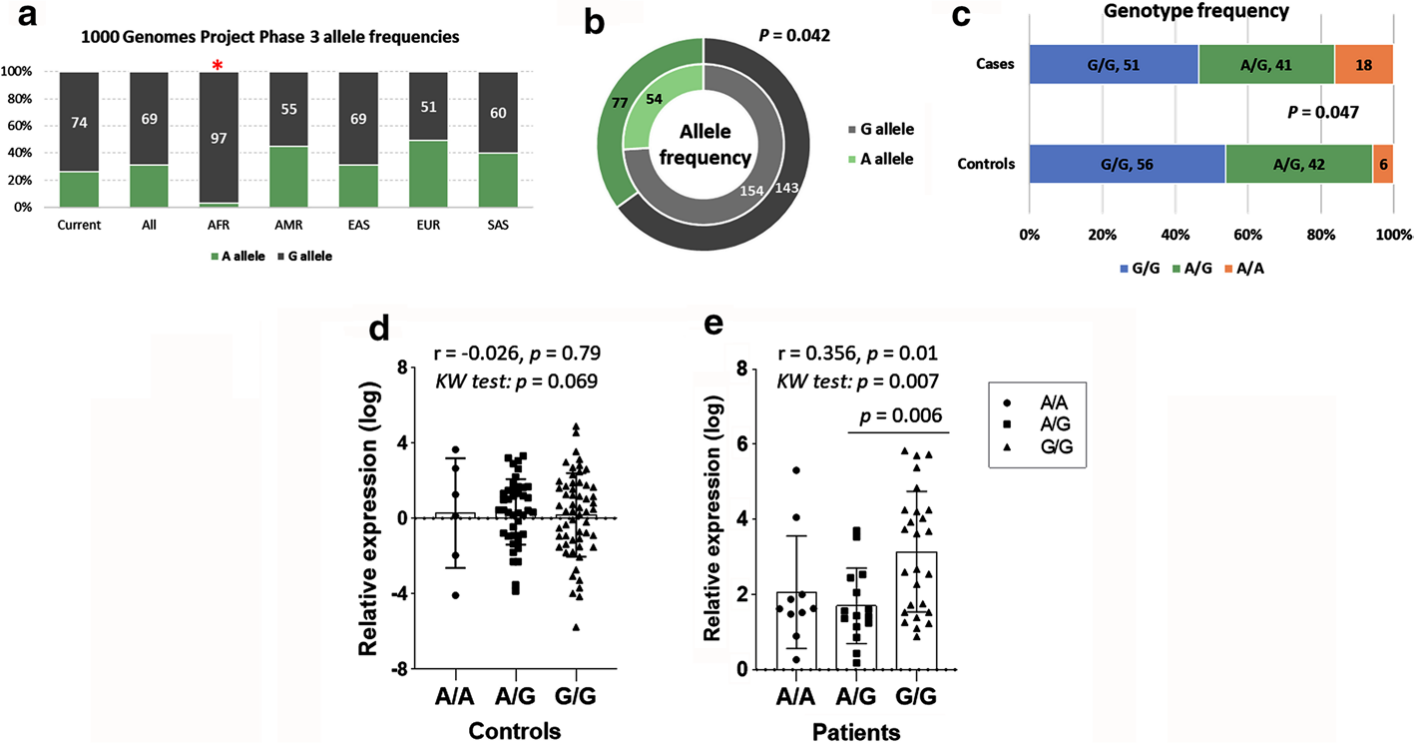
Genotype and allele frequencies of *CDKN2B-AS1* (rs2383207) variant and association with gene expression. **a**–**c** A two-sided Chi-square test was used. **d**, **e** Spearman’s correlation analysis was done, revealing the coefficient correlation value (r) and its *P*-value. Kruskal–Wallis (KW) test was applied to test for significant difference between cohorts with genotypes. Multiple comparison test was applied for pairwise comparison. Statistical significance was set at *P*-value < 0.05

A total of 110 cases and 104 controls were genotyped. Genotype frequencies followed the Hardy–Weinberg equilibrium in controls (*P* = 0.80) and cases (*P* = 0.06). Minor allele frequency (A allele) was 31% in the overall population and 26% in controls. Apart from the African population, allele frequencies were similar to those of different populations in the 1000 Genome Project Phase 3 (http://ensembl.org) (Fig. 2a). In our study population, half of the patients carried G/G genotype (107 cohorts), while A/G heterozygotes exceed a third (83 patients, 39%). A higher frequency of the A allele was observed in BC patients (77, 35%) compared to controls (54, 26%), *P* = 0.042 (Fig. 2b). Similarly, A/A homozygosity was the most frequent genotype among patients, representing 16% (18 patients) compared to 6% (6 patients), *P* = 0.047 (Fig. 2c).

#### CDKN2B-AS1 (rs2383207) genotypes and disease risk

According to the genetic association models, A allele (A/A) homozygotes were three times more liable to develop BC under homozygote comparison (OR = 3.27, 95% CI 1.20–8.88, *P* = 0.044) and the recessive model (OR = 3.17, 95% CI 1.20–8.34, *P* = 0.013) (Table 1).

**Table 1:** Genetic association models and disease risk

Model	Genotypes	Controls	Patients	Adjusted OR (95% CI)	*P*-value
Codominant	G/G	56 (53.9%)	51 (46.4%)	1.00	**0.044**
	A/G	42 (40.4%)	41 (37.3%)	1.07 (0.60–1.90)	
	A/A	6 (5.8%)	18 (16.4%)	**3.27 (1.20–8.88)**	
Dominant	G/G	56 (53.9%)	51 (46.4%)	1.00	0.28
	A/G–A/A	48 (46.1%)	59 (53.6%)	1.35 (0.79–2.30)	
Recessive	G/G–A/G	98 (94.2%)	92 (83.6%)	1.00	**0.013**
	A/A	6 (5.8%)	18 (16.4%)	**3.17 (1.20–8.34)**	
Over-dominant	G/G–A/A	62 (59.6%)	69 (62.7%)	1.00	0.64
	A/G	42 (40.4%)	41 (37.3%)	0.88 (0.51–1.52)	
Log-additive	–	–	–	1.48 (0.99–2.22)	0.053

Data are presented as number (percentage). Adjusted odds ratio (OR) by age. Bold values indicate statistical significance at *P*-value < 0.05

*CI* confidence interval

### *CDKN2B-AS1* genotypes modulate lncRNA expression in the breast cancer cohort

Although different *CDKN2B-AS1* genotypes in the control group showed no significant difference in gene relative expression (*P* = 0.069) (Fig. 2d), the G/G genotype was associated with a higher expression level than A/G in the BC cohort; median and quartile values were 3.14 (1.52–4.25) in G/G vs. 1.42 (0.93–2.35) in A/G and 1.62 (1.33–2.51) in A/A (*P* = 0.006) (Fig. 2e).

### Association of *CDKN2B-AS1* with clinicopathological features

Apart from body weight, in which overweight and obesity were more prevalent among G/G cohorts (*P* = 0.004), no significant differences in BC risk factors among patients with different genotypes were found (Table 2).

**Table 2:** Association between clinicopathological characteristics of breast cancer patients and *CDKN2B-AS1* genotypes

Characteristics	Total	A/A	A/G	G/G	*P*-value
Demographics and risk factors					
Age (years)					
≤ 45	98 (48.3)	8 (44.4)	24 (58.5)	26 (51)	0.38
> 45	105 (51.7)	10 (55.6)	17 (41.5)	25 (49)	
Smoking					
Negative	99 (90)	18 (100)	37 (90.2)	44 (86.3)	0.248
Positive	11 (10)	0 (0)	4 (9.8)	7 (13.7)	
Weight					
Underweight	12 (10.9)	4 (22.2)	4 (9.8)	4 (7.8)	**0.004**
Normal	28 (25.5)	7 (38.9)	13 (31.7)	8 (15.7)	
OW/Obese	70 (63.6)	7 (38.9)	24 (58.5)	39 (76.5)	
FH cancer					
Negative	76 (69.1)	13 (72.2)	28 (68.3)	35 (68.6)	0.951
Positive	34 (30.9)	5 (27.8)	13 (31.7)	16 (31.4)	
Breast problems					
Negative	102 (92.7)	18 (100)	38 (92.7)	46 (90.2)	0.387
Positive	8 (7.3)	0 (0)	3 (7.3)	5 (9.8)	
OCP intake					
Negative	87 (79.1)	17 (94.4)	29 (70.7)	41 (80.4)	0.114
Positive	23 (20.9)	1 (5.6)	12 (29.3)	10 (19.6)	
Menarche					
Late onset	39 (35.5)	4 (22.2)	11 (26.8)	24 (47.1)	0.058
Early onset	71 (64.5)	14 (77.8)	30 (73.2)	27 (52.9)	
Parity					
Multipara	96 (87.3)	14 (77.8)	37 (90.2)	45 (88.2)	0.401
Nullipara	14 (12.7)	4 (22.2)	4 (9.8)	6 (11.8)	
Gravida					
Early G1	105 (95.5)	18 (100)	38 (92.7)	49 (96.1)	0.443
Late G1	5 (4.5)	0 (0)	3 (7.3)	2 (3.9)	
Menopause					
Early	98 (89.1)	16 (88.9)	37 (90.2)	45 (88.2)	0.953
Late	12 (10.9)	2 (11.1)	4 (9.8)	6 (11.8)	
Breast feeding					
Negative	92 (83.6)	13 (72.2)	34 (82.9)	45 (88.2)	0.284
Positive	18 (16.4)	5 (27.8)	7 (17.1)	6 (11.8)	
Menopausal status					
Pre	80 (72.7)	14 (77.8)	28 (68.3)	38 (74.5)	0.698
Post	30 (27.3)	4 (22.2)	13 (31.7)	13 (25.5)	
Night work					
Negative	97 (88.2)	16 (88.9)	36 (87.8)	45 (88.2)	0.993
Positive	13 (11.8)	2 (11.1)	5 (12.2)	6 (11.8)	
Sedentary life style					
Negative	11 (10)	1 (5.6)	3 (7.3)	7 (13.7)	0.470
Positive	99 (90)	17 (94.4)	38 (92.7)	44 (86.3)	
Pathological data					
Side					
Right	70 (63.6)	11 (61.1)	27 (65.9)	32 (62.7)	0.92
Left	40 (36.4)	7 (38.9)	14 (34.1)	19 (37.3)	
Site					
Outer quadrants	48 (43.6)	5 (27.8)	16 (39)	27 (52.9)	0.13
Others	62 (56.4)	13 (72.2)	25 (61)	24 (47.1)	
No masses					
Single	85 (77.3)	12 (66.7)	30 (73.2)	43 (84.3)	0.22
Multiple	25 (22.7)	6 (33.3)	11 (26.8)	8 (15.7)	
Grade					
≤ 2	90 (81.8)	16 (88.9)	32 (78)	42 (82.4)	0.60
> 2	20 (18.2)	2 (11.1)	9 (22)	9 (17.6)	
T stage					
≤ 3	79 (71.8)	12 (66.7)	29 (70.7)	38 (74.5)	0.80
> 3	31 (28.2)	6 (33.3)	12 (29.3)	13 (25.5)	
N stage					
N0	30 (27.3)	8 (44.4)	10 (24.4)	12 (23.5)	0.20
N1–3	80 (72.7)	10 (55.6)	31 (75.6)	39 (76.5)	
M stage					
M0	50 (45.5)	8 (44.4)	17 (41.5)	25 (49)	0.76
M1	60 (54.5)	10 (55.6)	24 (58.5)	26 (51)	
LVI					
Negative	58 (52.7)	10 (55.6)	18 (43.9)	30 (58.8)	0.35
Positive	52 (47.3)	8 (44.4)	23 (56.1)	21 (41.2)	
Skin involvement					
Negative	89 (80.9)	14 (77.8)	31 (75.6)	44 (86.3)	0.40
Positive	21 (19.1)	4 (22.2)	10 (24.4)	7 (13.7)	
Clinical stage					
≤ 2	48 (43.6)	8 (44.4)	17 (41.5)	23 (45.1)	0.93
> 2	62 (56.4)	10 (55.6)	24 (58.5)	28 (54.9)	
NPI					
Good	56 (50.9)	12 (66.7)	19 (46.3)	25 (49)	0.332
Poor	54 (49.1)	6 (33.3)	22 (53.7)	26 (51)	
Molecular subtype					
Luminal A	52 (47.3)	9 (50)	16 (39)	27 (52.9)	0.45
Luminal B	14 (12.7)	3 (16.7)	3 (7.3)	8 (15.7)	
HER2^+^	7 (6.4)	1 (5.6)	4 (9.8)	2 (3.9)	
Basal	37 (33.6)	5 (27.8)	18 (43.9)	14 (27.5)	
IHPI					
Good	66 (181.6)	12 (66.7)	19 (46.3)	35 (68.6)	0.25
Moderate	37 (33.6)	5 (27.8)	18 (43.9)	14 (27.5)	
Poor	7 (6.4)	1 (5.6)	4 (9.8)	2 (3.9)	
Follow-up					
ESMO					
Low risk	41 (37.3)	9 (50)	16 (39)	16 (31.4)	0.35
High risk	69 (62.7)	9 (50)	25 (61)	35 (68.6)	
DFS					
Prolonged	52 (47.3)	7 (38.9)	13 (31.7)	32 (62.7)	**0.009**
Short	58 (52.7)	11 (61.1)	28 (68.3)	19 (37.3)	
Recurrence					
Negative	55 (50)	9 (50)	19 (46.3)	27 (52.9)	0.82
Positive	55 (50)	9 (50)	22 (53.7)	24 (47.1)	
OS					
Prolonged	53 (48.2)	2 (11.1)	8 (19.5)	43 (84.3)	**< 0.001**
Short	57 (51.8)	16 (88.9)	33 (80.5)	8 (15.7)	

Data are presented as numbers (percentage). A two-sided Chi-square test was used. Short survival was set at 12 months. Bold values indicate significance at *P*-value < 0.05

*OW* over-weight, *FH* family history, *OCP* oral contraceptive pills, *G1* first pregnancy, *LVI* lymphovascular invasion, *NPI* Nottingham prognostic index, *IHPI* immunohistochemistry prognostic index, *ESMO* European Society of Medical Oncology for risk estimation of recurrence, *DFS* disease-free survival, *OS* overall survival

**Fig. 3:**
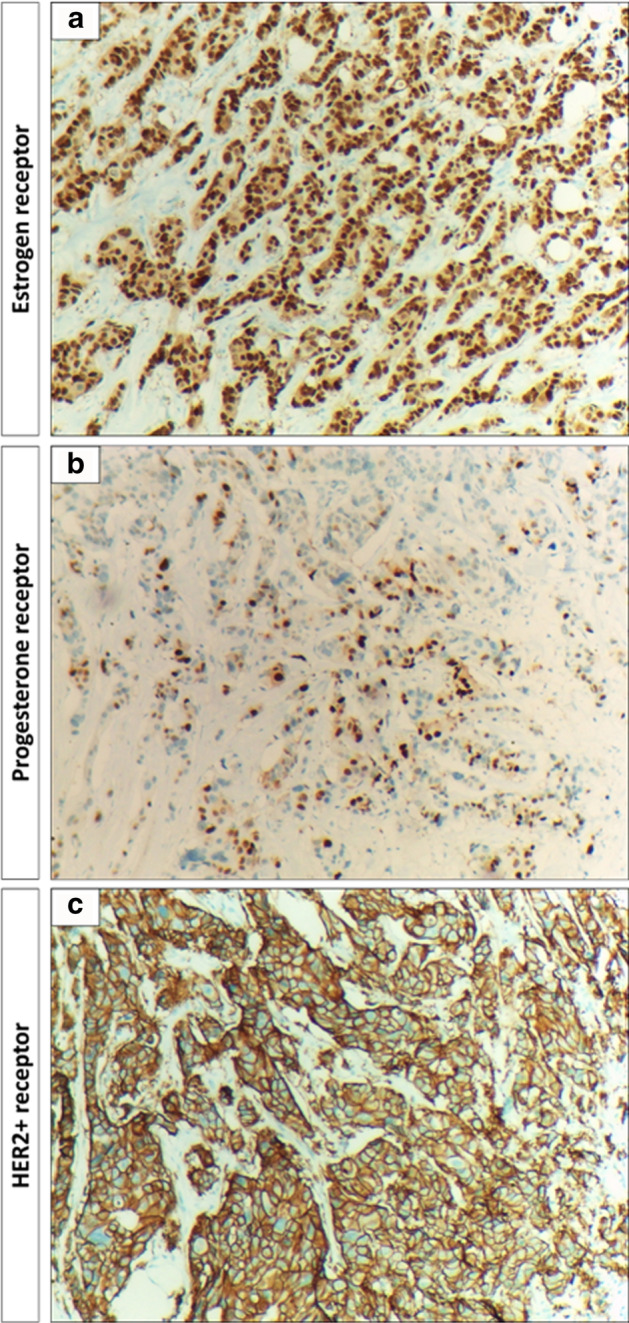
Immunohistochemical analysis of breast cancer samples. **a** Infiltrating lobular carcinoma at 10× magnification power showing that tumor cells are markedly positive for estrogen hormonal nuclear receptors in about 85% of tumor cells, score 3+ 5/8. **b** Infiltrating lobular carcinoma of the breast at 10× magnification power showing that tumor cells are moderately positive for progesterone hormonal nuclear receptors in about 35% of tumor cells, score 2+ 4/8. **c** Infiltrating duct carcinoma at 10× magnification power showing that tumor cells are markedly positive staining with continuous and intense membranous staining for Her2^+^ protein, over-expression score + 3

Immunohistochemical analysis was conducted for tumor samples. Selected samples with positive estrogen, progesterone, and HER2^+^ receptors are illustrated in Fig. 3.

**Fig. 4:**
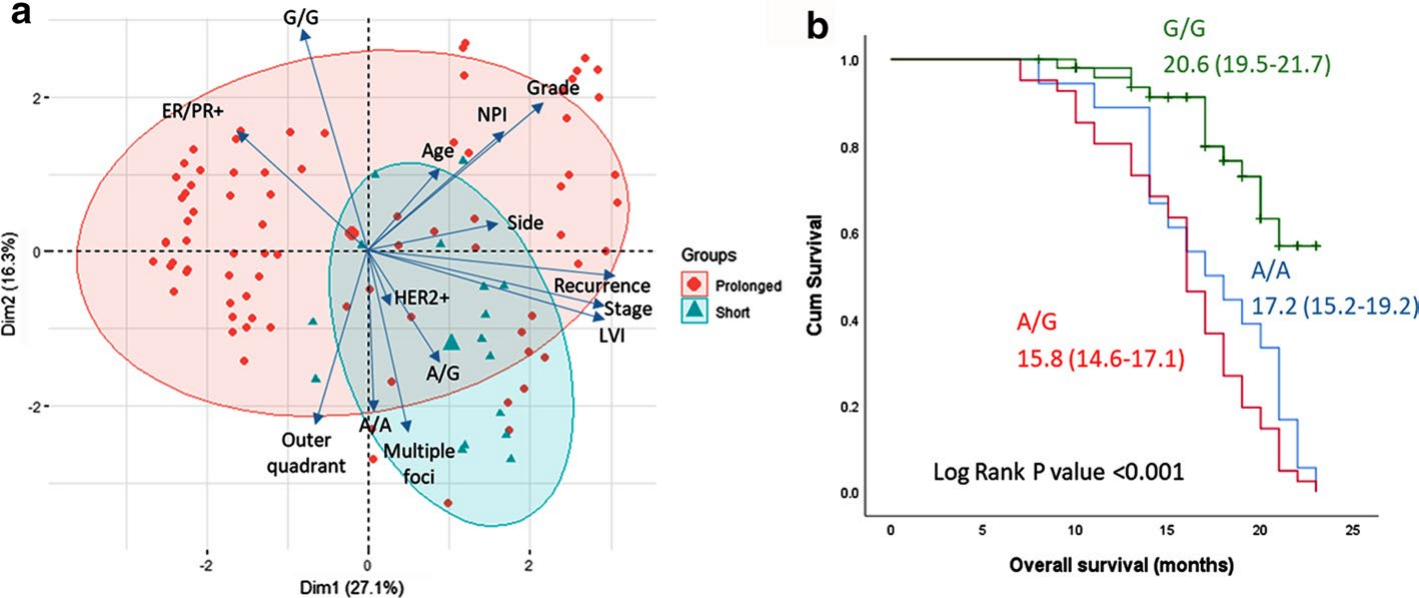
Impact of *CDKN2B-AS1* variant on survival. **a** Principal component analysis plots the interactions of variables. Each arrow represents a variable with a longer length indicating a greater impact on distribution of the samples. Each observation is symbolized as a triangle (if it has short overall survival) or circle (if it has prolonged survival). Mortality was associated with A/A and A/G genotypes. They were more likely to have ER/PR− and HER2^+^ receptors and multiple foci. **b** Kaplan–Meier curve showing the mean and confidence interval duration of overall survival according to their genotype status. G/G homozygote patients had longer survival than their counterparts (*P* < 0.001)

Regarding the pathological characteristics of BC tumors, no significant difference was observed among patients with various genotypes. However, the rs2383207*A variant was associated with poor survival. Higher frequencies of A/A and A/G genotypes were found in cohorts with short disease-free survival (*P* = 0.009) and overall survival (*P* < 0.001) (Table 2). Consistently, the principal component analysis showed a deviation of patients who had shorter survival towards A/A and A/G genotypes, multiple lesions, advanced stage, lymphovascular invasion, and HER2^+^ receptor staining (Fig. 4a). Kaplan–Meier curve analysis also revealed a higher mean survival duration of G/G cohorts (20.6 months) compared to their counterparts (A/A: 15.8 months and A/G: 17.2 months), *P* < 0.001 (Fig. 4b).

**Fig. 5:**
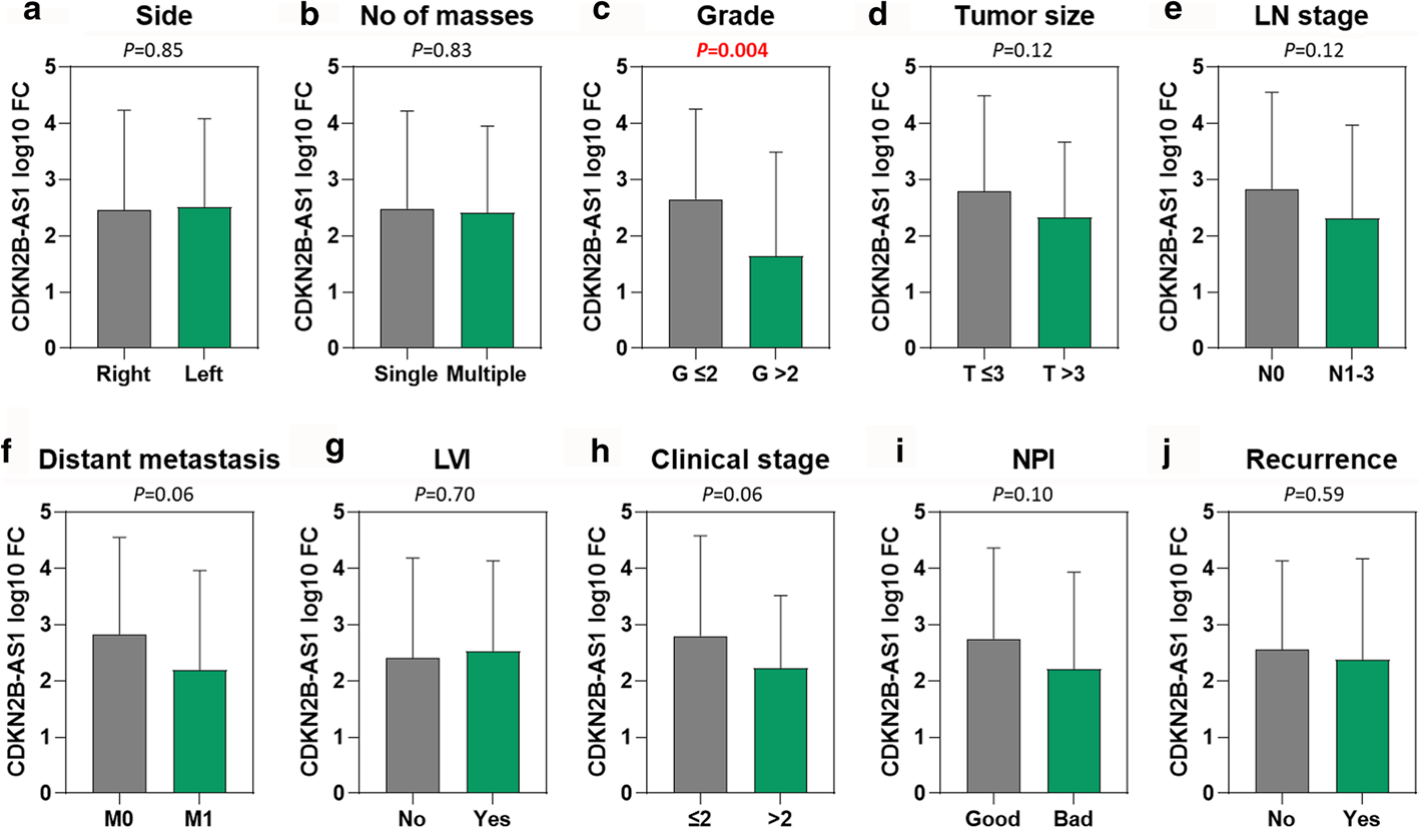
Association of *CDKN2B-AS1* gene expression level with clinical and pathological parameters. **a** Side of the BC. **b** Number of BC masses. **c** BC grade. **d** BC tumor size. **e** Lymph node stage. **f** Distant metastasis. **g** Lymphovascular infiltration. **h** Clinical stage of BC patients. **i** Nottingham prognostic index for BC patients. **j** Recurrence of BC patients. Fold change was log10 transformed. Student’s t-test was used. *P*-values below 0.05 were set to be significant. *OQ* outer quadrant, *LN* lymph node, *LVI* lymphovascular infiltration, *NPI* Nottingham prognostic index

In an association of the transcriptomic signature of *CDKN2B-AS1* with clinicopathological features, the lower expression level (1.64 ± 1.85) was associated with poor pathological grade compared to well-differentiated and intermediately differentiated tumors (2.65 ± 1.60, *P* = 0.004) in breast cancer patients. However, there were no other significant associations with any other clinical or pathological parameters (Fig. 5).

### In silico data analysis

#### Structural analysis of *CDKN2B-AS1* gene

Although the *CDKN2B-AS1* gene locus includes other protein-coding genes, i.e. *CDKN2B* (encoding p15ink4b) and *CDKN2A* (encoding p16ink4a and p14ARF) (Fig. 1b) with a key role in cell cycle inhibition, senescence, and stress-induced apoptosis, prior studies highlighted that SNPs in this locus act through effects on CDKN2B-AS1 itself. The gene has 28 splice variant linear and circular transcripts ranging from 602 bp up to 7173 bp and includes LINE, SINE, and Alu repetitive sequences. Some are tissue-specific, while others have distinct roles in cell physiology.

#### Variant analysis of *CDKN2B-AS1* gene

The studied intronic variant rs2383207A>G in the *CDKN2B-AS1* gene is located at chromosome 9: 22115959 (forward strand) 2161 bp away from the splice site with a minor allele frequency (G allele) of 0.31. This SNP overlaps 15 transcripts of the lncRNA and is predicted to be benign (Additional file 1: Table S2). Common disease genome-wide association studies (GWAS) have identified the *CDKN2B-AS1* gene as a shared locus for genetic susceptibility to multiple cancers (Fig. 1d).

#### Transcriptomic profile of *CDKN2B-AS1* gene

**Fig. 6:**
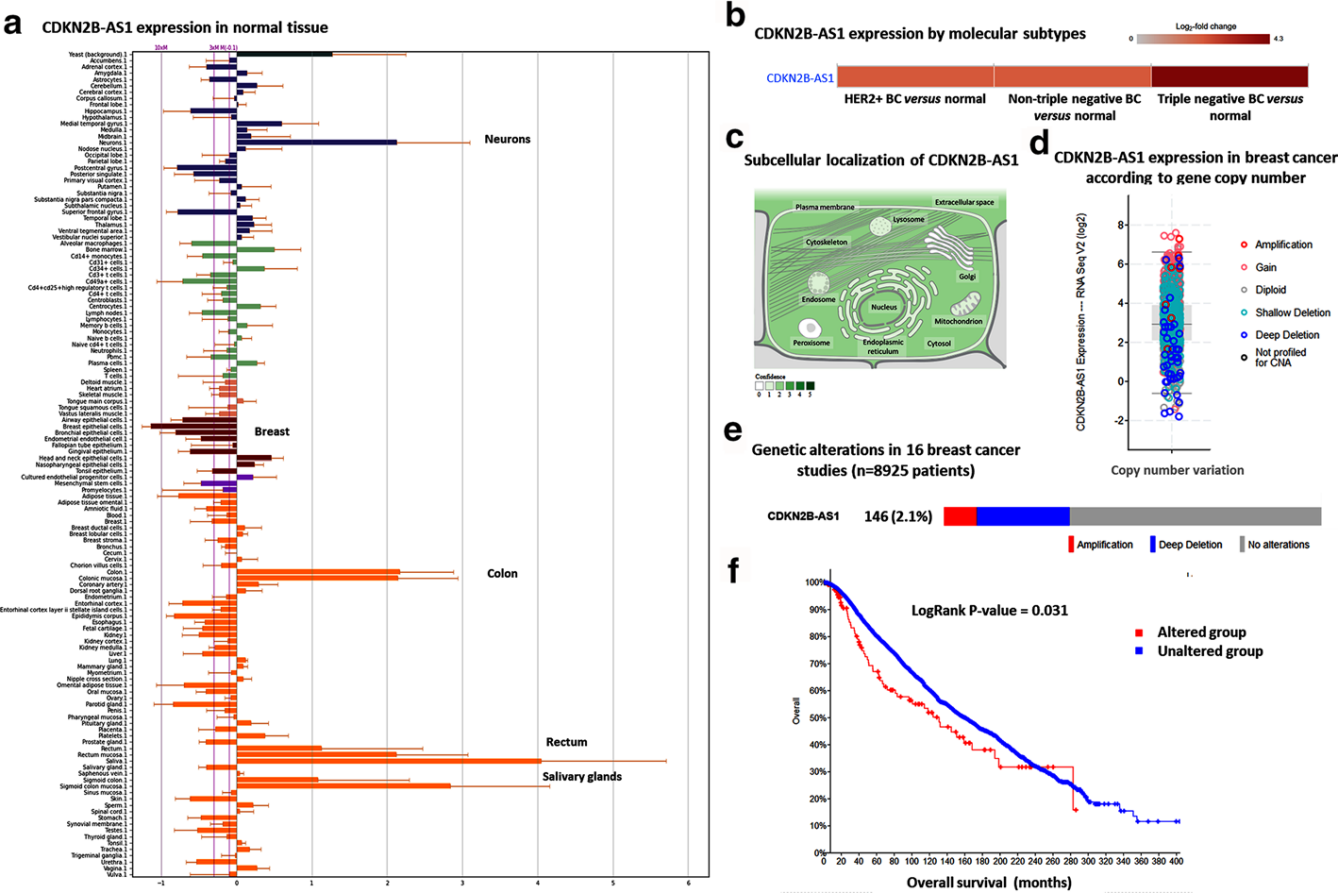
CDKN2B-AS1 expression in normal and cancer states. **a** lncRNA expression in 27 normal tissues from GTEx Portal showed upregulation of CDKN2B-AS1 in colorectal tissue and salivary glands followed by neurons while being down-regulated in breast tissue. **b** Expression level of lncRNA in breast cancer tissues with different receptor status. **c** Subcellular localization of CDKN2B-AS1. **d**–**f** Analysis of 8925 breast cancer patients in the TCGA across 16 studies. **d** Expression level of *CDKN2B-AS1* according to the gene copy number. **e** One hundred forty-six patients (2.1%) had a genetic alteration in the *CDKN2B-AS1* gene (44 amplifications and 102 deep deletions). **f** Patients with altered *CDKN2B-AS1* had poor survival

RNAseq analysis of 27 different normal tissues showed higher expression of the colon and small intestine and down-regulation in normal breast tissues (Fig. 6a). However, in BC tissues, overexpression was observed in all molecular subtypes (Fig. 6b). *CDKN2B-AS1* accumulates in both the nucleus and cytoplasm. The family of linear *CDKN2B-AS1* contains proximal (exon 1) and distal (exon 13b, 19) exons, and is enriched in the nucleus. However, the circular isoforms usually contain the middle exons (exons 5, 6, and 7), are enriched in the cytoplasm, and they differed markedly in their stability. Circular lncRNAs were reported to be associated with ribosome biogenesis and nucleolar stress, while nuclear isoforms are more likely to be involved in regulating gene transcription via chromatin modulation (Fig. 6c). Sixteen BC studies from the TCGA including 8925 patients were retrieved. Of these, 146 patients (2.1%) had a genetic alteration in the *CDKN2B-AS1* gene (44 amplification and 102 deep deletions). Somatic mutations were not studied in these datasets. Higher expression levels were encountered in patients with more copies of the genes and vice versa. Patients with altered *CDKN2B-AS1* had poorer survival than their counterparts (*P* = 0.031) (Fig. 6d–f).

#### Prognostic and predictive role of *CDKN2B-AS1* gene in breast cancer

**Fig. 7:**
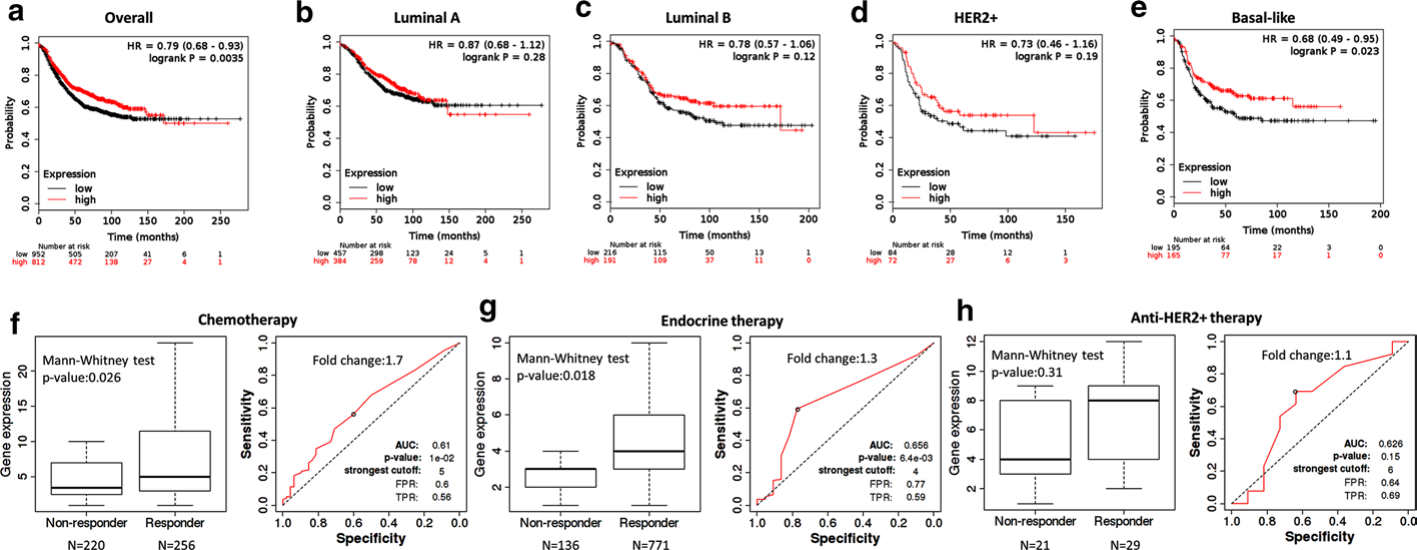
Prognostic and predictive role of *CDKN2B-AS1* gene in breast cancer (BC). **a**–**e** Kaplan–Meier plots for BC patients according to their molecular subtypes (Data source: KMplotter). **a** Overall analysis. **b** Luminal A. **c** Luminal B. **d** HER2^+^. **e** Basal-like BC subtype. **f**–**h** Comparison of *CDKN2B-AS1* expression level between responder and non-responder and analysis of relapse-free survival at 5 years. (data source: http://rocplot.org) in BC patients receiving different therapeutic modalities. **f** BC patients receiving chemotherapy. **g** BC patients receiving endocrine therapy. **h** BC patients receiving anti-HER2^+^ therapy

**Fig. 8:**
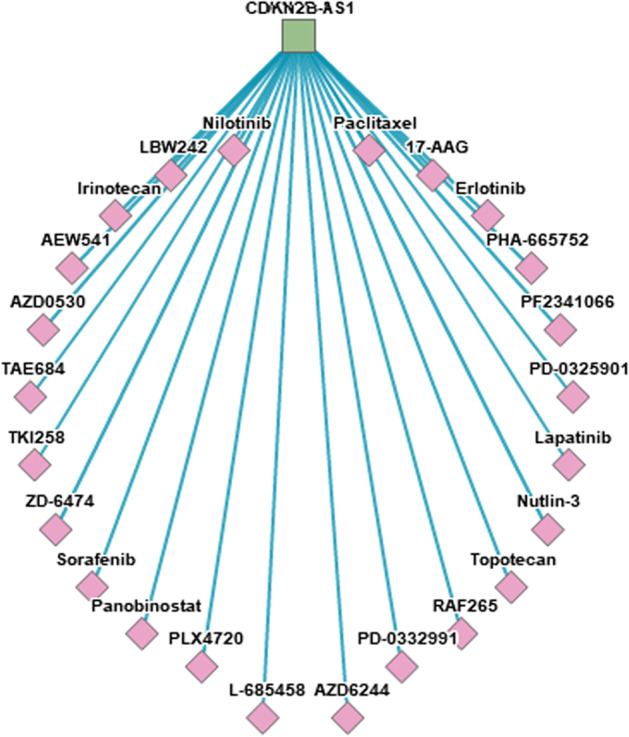
Drug-LncRNA Network. The top 30 drug-lncRNA pairs were used to build the network. The square and the diamond represent lncRNA and drug, respectively (Data source: LncMap (http://bio-bigdata.hrbmu.edu.cn/LncMAP/drug_lnc.jsp))

Despite the up-regulation of the lncRNA in BC tissues and blood, a higher expression level of the gene was associated with better overall survival (Fig. 7a–e). The prognostic performance of lncRNA expression, represented as a ROC curve, is shown in Fig. 7f–h. The utility of *CDKN2B-AS1* as a diagnostic and prognostic biomarker is illustrated in Table 3. Articles showing the association of *CDKN2B-AS1* up-regulation with treatment resistance are shown in Table 4. The top 30 drug-lncRNA pairs were used to build the Drug-LncRNA Network. Of the drugs associated with *CDKN2B-AS1*, Lapatinib, a dual inhibitor of EGFR and HER2, is indicated for patients with advanced or metastatic BC treatment whose tumors overexpress HER2. Other tyrosine kinase inhibitors such as erlotinib and sorafenib were also connected to the lncRNAs. The single drug topotecan was also indicated in patients with BC (Fig. 8).

**Table 3:** CDKN2AB-AS1 quantification in cancer patients as a diagnostic and prognostic biomarker. *Data source*: Lnc2Cancer version 2.0

Cancer type	Methods	Expression pattern	PubMed ID
Circulatory diagnostic biomarker			
** Breast cancer**	**Microarray, qPCR**	Up-regulated	**28248879**
Intraductal Papillary Mucinous Neoplasms of the Pancreas	qPCR	Up-regulated	28874676
Acute lymphoblastic leukemia	MassARRAY assay	Up-regulated	21414664
Neurofibromatosis type 1	Microarray, qPCR	Up-regulated	22034633
Non-small cell lung cancer	qPCR	Up-regulated	29504701
Oral cancer	qPCR, a Luciferase reporter assay, in vitro knockdown	Up-regulated	29635126
Prognostic biomarker			
** Breast cancer**	**Microarray, qPCR**	**Up-regulated**	**28248879**
** Triple-negative breast cancer**	**qPCR, Luciferase reporter assay, RIP**	**Up-regulated**	**28961506**
Non-small cell lung cancer	qPCR	Up-regulated	29504701
Oral cancer	qPCR, a Luciferase reporter assay, in vitro knockdown	Up-regulated	29635126
Gastric cancer	qPCR, RNAi	Up-regulated	27121324
Lung adenocarcinoma	qPCR, Western blot	Up-regulated	28402932
Bladder cancer	qPCR, RNAi, Western blot	Up-regulated	26800519
Cervical cancer	qPCR, RNAi, Western blot, Cell proliferation assay	Up-regulated	27899255
Colorectal cancer	qPCR, RNAi, Western blot, Northern blot	Up-regulated	27286457
Colorectal cancer	qPCR, RNAi	Up-regulated	27314206
Esophageal squamous cell cancer	qPCR, RNAi, Western blot	Up-regulated	24747824
Gallbladder cancer	qPCR, Western blot	Down-regulated	26812694
Gastric cancer	qPCR, RNAi, Western blot, RIP	Up-regulated	24810364
Gastric cancer	qPCR, RNAi, Western blot, a Luciferase reporter assay, Cell proliferation assay	Up-regulated	27027260
Glioblastoma	qPCR	Up-regulated	23046790
Hepatocellular carcinoma	qPCR, Western blot	Up-regulated	29029488
Hepatocellular carcinoma	qPCR, RNAi	Up-regulated	26045820
Lung cancer	qPCR, RNAi, Western blot, Cell apoptosis assay	Up-regulated	25964559
Nasopharyngeal cancer	RT-qPCR, Western blot, a Luciferase reporter assay, in vitro knockdown, RIP	Up-regulated	29463902
Nasopharyngeal cancer	qPCR, RNAi, Western blot, MTT assay	Up-regulated	27557514
Non-small cell lung cancer	qPCR, RNAi, Western blot, Luciferase reporter assay	Up-regulated	27307748
Non-small cell lung cancer	qPCR, RNAi, Cell proliferation assay	Up-regulated	25889788
Non-small cell lung cancer	qPCR, Western blot	Up-regulated	25504755
Osteosarcoma	qRT-PCR, Western blot, in vitro knockdown	Up-regulated	29520337
Ovarian cancer	qPCR, Western blot	Up-regulated	27095571
Pancreatic cancer	qPCR, Cell transfection, Western blot, cell migration, and invasion assay	Up-regulated	28344092
Renal cell carcinoma	qPCR, RNAi, Western blot, Cell migration and invasion assay, CCK-8 assay	Up-regulated	28251886
Serous ovarian cancer	qPCR, RNAi, Western blot	Up-regulated	25845387
Thyroid cancer	qPCR, RNAi, Western blot	Up-regulated	27507052

Breast cancer-related articles are shown in bold

**Table 4:** Publications on the role of *CDKN2B-AS1* with treatment resistance in cancer

Cancer site	Study type	Treatment	Putative function	Putative mechanism	PubMed ID
Osteosarcoma	In vitro	Cisplatin	Chemoresistance	Through STAT3 and miR-125a-5p	30777616
Colorectal cancer	In vitro	5-Fluorouracil	Chemoresistance	by regulating ATP-binding cassette subfamily C member 1 through binding Let-7a	30279206
Lung adenocarcinoma	In vitro	Paclitaxel	Chemoresistance	Through the mitochondrial pathway by modulating the expression of apoptosis-related protein cleaved-PARP and Bcl-2	28402932
Nasopharyngeal carcinoma	In vitro	Radiation	Radioresistance	Via functioning as a miR-125a sponge	28402230
	In vitro	Cisplatin	Chemoresistance	Via regulating microRNA let-7a	28117929
Multiple myeloma	Patients	Hematopoietic stem cell transplantation	Relapse	By modulating p14ARF-MDM2-p53 axis	28150872
Gastric cancer	In vitro	Cisplatin and 5-fluorouracil	Multidrug resistance	Decreased the expression of MDR1 and MRP1	27121324
Bladder cancer		Gemcitabine	Resistance	Through the Wnt signaling pathway	29937935
laryngeal squamous cell cancer		Cisplatin and paclitaxel		CDKN2B-AS1 expression decrease	25257554
Oral squamous cancer		Cisplatin	Resistance	Via impairment of the drug transporters MRP1 and ABCC2	29176691

#### Functional enrichment analysis of *CDKN2B-AS1* gene

**Fig. 9:**
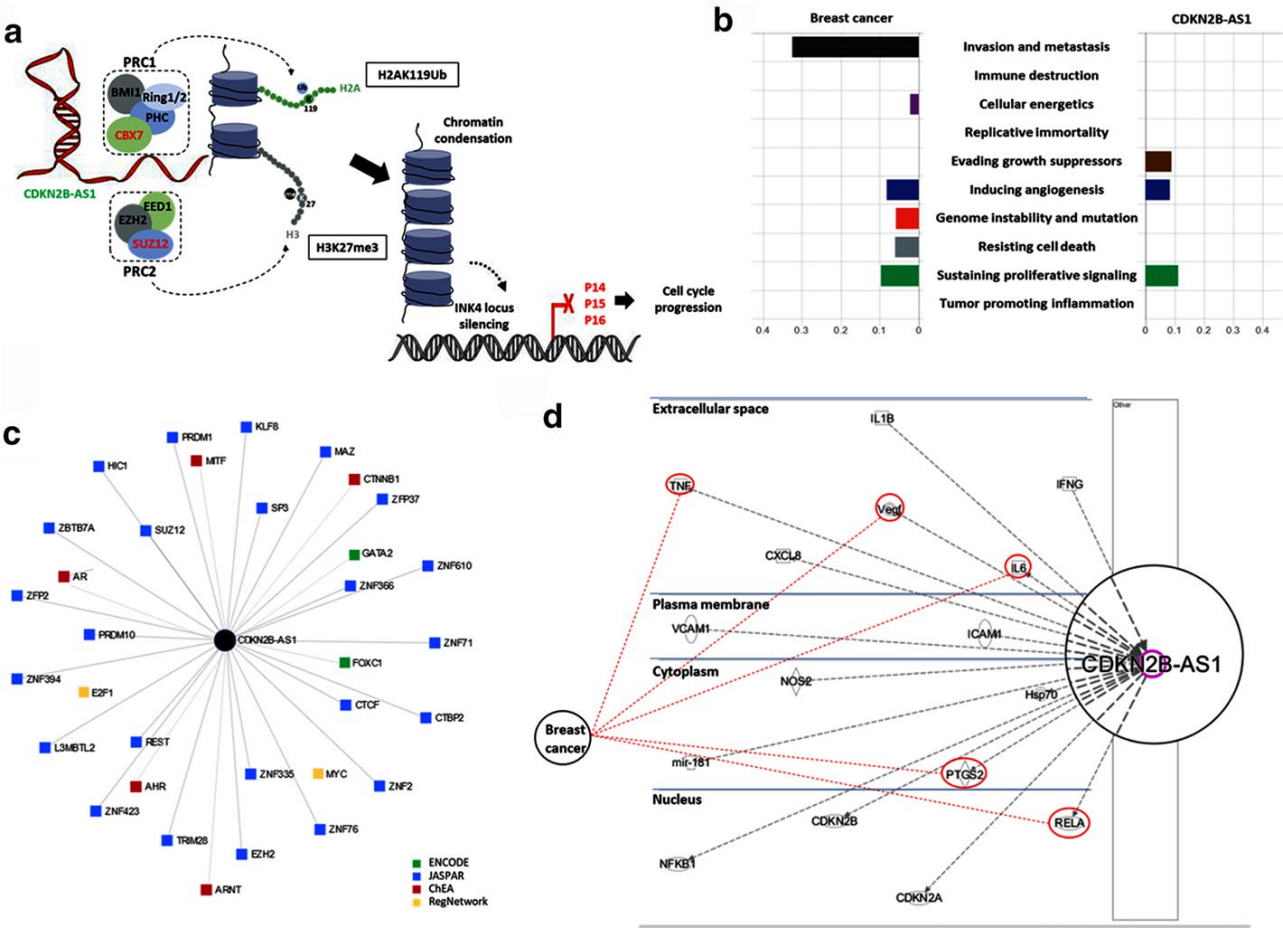
Functional role of CDKN2B in cancer. **a** In cis and in trans gene regulation of *CDKN2B-AS1* through chromatin modification complexes. CDKN2B-AS1 acts as docking for the epigenetic regulators, PRC1 and PRC2 polycomb complexes, to modify the histone code. The PRC1 complex includes multiple subunits, such as PHC, CBX7, BMI1, and RING1a/1b, which are implicated in the maintenance of silencing by catalyzing monoubiquitination of histone H2A (H2AK119ub1). The PRC2 complex comprises the JARID2, EED, SUZ12, and EZH2 subunits which maintain chromatin repression by catalyzing mono-/di-/trimethylation of histone H3 lysine 27 (H3K27me1, H3K27me2, and H3K27me3). **b** Automatic CHAT classification of the PubMed literature according to the hallmark of cancer taxonomy. Breast cancer and CDKN2B-AS1 (data are shown as NPMI; normalized pointwise mutual information) were compared. Each bar demonstrates cancer hallmark and/or biological process association with the search query. **c** Transcription factors-gene network in Network Analyst. A total of 34 nodes representing TF-gene interactions with the lncRNA CDKN2B-AS1 and edges with length representing the degree of association score. Different databases were used: ENCODE, JASPAR, ChEA, and RegNetwork. **d** Ingenuity Pathway Analysis. A gene network was built to connect key genes highly enriched with the lncRNA. The direction of activity (activation/inhibition) is shown. Five molecular targets involved in breast cancer are further connected in red

Functional enrichment analysis revealed *CDKN2B-AS1* as a key player in regulating gene expression and cell migration. Being within the *INK4b-ARF-INK4a* gene family, which encodes the p15, p14, and p16 tumor-suppressor proteins, respectively, it is transcriptionally silenced or homozygously deleted in various human cancers. These three proteins are implicated in apoptosis, senescence, and stem cell renewal via promoting anti-proliferative and pro-apoptotic activities of Rb1 and p53 (Fig. 9a).

We used CHAT to analyze PubMed literature on BC and our studied gene. Cell invasion and metastasis was the most common hallmark associated with BC literature, followed by sustaining proliferative signaling. *CDKN2B-AS1* studies have two main hallmarks similar to those of the BC profile: sustaining proliferative signaling and genome instability and mutation (Fig. 9b). Upstream regulators enhancing transcription of the *CDKN2B-AS1* gene are presented in Fig. 9c. The LncMAP revealed that CDKN2B-AS1 could also bind to 12 TFs: E2F4, REST, BCL11A, RARA, ETS1, ESR2, GATA6, FLI1, EBF1, CTNNB1, STAT2, and ESRRA.

As depicted in Fig. 9d, Ingenuity Pathway Analysis showed key genes highly enriched in BC with *CDKN2B-AS1*, namely (1) *RELA* proto-oncogene, (2) prostaglandin-endoperoxide synthase 2 (*PTGS2*), (3) interleukin 6 (*IL6*), (4) vascular endothelial growth factor A (*VEGFA*), and (5) tumor necrosis factor-alpha (*TNF*).

## Discussion

The last two decades have witnessed remarkable developments in the era of non-coding RNAs [40]. Given their high stability in different storage and handling conditions, being easily monitored by repeated sampling and their circulatory levels mirroring those in tissue cancer, serum/plasma lncRNAs demonstrated features of particular relevance for ideal biomarkers [41].

In this study, we evaluated the impact of serum lncRNA CDKN2B-AS1 expression and variant signature in BC patients supported by in silico analyses followed by verifying the results on samples from TCGA. We found that CDKN2B-AS1 is upregulated in sera of BC patients compared with controls. This observation was in line with the oncogenic role this lncRNA plays in several cancers (Additional file 1: Table S3), which can support the potential role this type of lncRNA can play as a universal biomarker differentiating cancer from non-cancer patients.

CDKN2B-AS1 is known to be involved in transcriptional repression through forming chromatin modification complexes that execute histone modifications at specific sites. It can interact with both chromobox 7 (CBX7), a polycomb repressor component within PRC-1, and SUZ12, a subunit of PRC2 [42]. Next, PRC2 downregulates *INK4* expression (Fig. 9a) by inducing H3K27 tri-methylation. Meanwhile, PRC1 maintains the repressive chromatin structure by H2AK119 mono-ubiquitination [15, 43].

*CDKN2B-AS1* is mainly upregulated by the E2F1 in an ATM-dependent manner after DNA damage, leading to cell cycle arrest to allow for DNA repair [44]. Several known potent oncogenes that regulate *CDKN2B-AS1* expression in various cancers have been reported in the literature, including MYC, RELA, and ERBB2 [12, 45]. *CDKN2B-AS1* expression is also regulated by interferon-gamma (IFNγ), tumor necrotic factor (TNFα) [46], and interleukin 1 beta (IL1B). These inflammatory mediators activate the nuclear factor kappa-B (NF-κB), a pro-proliferation and survival factor, pathway and form a complex with the YY1 transcription factor to create transcriptional regulatory loops [47].* CDKN2B-AS1* abundance can also be induced by exposure to hypoxia (a well-known phenomenon associated with the tumor microenvironment). It can bind the aryl hydrocarbon receptor nuclear translocator (ARNT), which functions as a transcriptional regulator of the adaptive response to hypoxia [48]. Apart from the potential oncogenic role of *CDKN2B-AS1* in BC, all the above mechanisms could support *CDKN2B-AS1* upregulation in breast cancer in part as an adaptive response to DNA and cellular damage during the tumorigenesis process, which could explain the association of high expression of this type of lncRNA with survival and cancer grade; the findings were validated by the same results from the TCGA data set. Interestingly, using RNAscope, a recent study underscored that presence of *CDKN2B-AS1* in different subcellular locations in breast tumors may affect its functionality in cancer progression [49].

Our in-silico* analysis*, including the Ingenuity Pathway Analysis, showed several key genes highly enriched with *CDKN2B-AS1* in BC and could mediate part of its oncogenic role or impact the tumor microenvironment in this type of cancer. These genes include (1) the *RELA* (v-rel avian reticuloendotheliosis viral oncogene homolog A) gene encoding the NF-κB-p65 subunit (a ubiquitous TF held in the cytoplasm in an inactive state by a specific inhibitor), upon degradation of which the NF-κB translocates to the nucleus and activates specific gene expression, (2) *PTGS2* encoding a major enzyme in prostaglandin (PTG) biosynthesis implicated in biosynthesis of prostanoids that are involved in inflammation and mitogenesis, (3) *IL6,* which functions in inflammation and B-cell maturation, (4) *VEGFA,* which stimulates proliferation and migration of vascular endothelial cells and is a key player in physiological/pathological angiogenesis, and (5) the *TNF* gene that regulates cell proliferation, differentiation, apoptosis, lipid metabolism, and coagulation.

It is worth noting that the transcriptomic abundance of *CDKN2B-AS1* is not only modulated by epigenetic control through promoter transcriptional activity, promoter methylation, and splicing [50] but also post-transcriptionally regulated by miRNA sponging and RNA stability [12].

Using the ENCORI database, we identified the crosstalk of CDKN2B-AS1 lncRNA with mRNAs and transcribed pseudogenes in cancer. Putative lncRNA–ceRNA interactions in cancer represent a new layer of gene regulation (Table 5). Mounting data from the literature suggest that deregulated CDKN2B-AS1–miRNA interacting networks have also been implicated in carcinogenesis, thus representing another molecular mechanism of the lncRNA. CDKN2B-AS1 contains miRNA-binding domains in its sequence and therefore acts as a "sponge" to sequester miRNAs away from its mRNA targets. Through modulation of microRNA pathways, CDKN2B-AS1 can also be involved in post-transcriptional regulation (Table 6). miRNA sponge mechanism was reported in BC patients with triple-negative receptors through interaction with microRNA-199a [51]. All the aforementioned cellular and genetic/epigenetic mechanisms support CDKN2B-AS1 involvement in breast carcinogenesis and suggest that it could be a biomarker for BC detection with other molecular panels.

**Table 5:** Crosstalk between the lncRNA (*CDKN2B-AS1*), mRNAs, and pseudogenes in competing endogenous RNA (ceRNA) network. *Data source*: ENCORI for RNA interactomes supported by CLIP-seq data (http://starbase.sysu.edu.cn/)

ceRNA symbol	ceRNA name	Molecule type	Location	ceRNA gene Type	FDR	Pan-cancer number
AL160290.2	Cytochrome *C* Oxidase Subunit VIIc (COX7C)	–	–	LincRNA	2.00E−03	14
RPL39P26	Ribosomal Protein L39 Pseudogene 26	–	–	Processed pseudogene	2.00E−03	8
CLCN1	Chloride Voltage-Gated Channel 1	Ion channel	Plasma membrane	Protein-coding	7.94E−04	17
ARHGAP44	Rho GTPase Activating Protein 44	Other	Cytoplasm	Protein-coding	7.94E−04	13
RBM6	RNA Binding Motif Protein 6	Other	Nucleus	Protein-coding	7.94E−04	19
AK9	Adenylate Kinase 9	Kinase	Nucleus	Protein-coding	8.97E−04	18
USP2	Ubiquitin Specific Peptidase 2	Peptidase	Cytoplasm	Protein-coding	8.78E−04	17
SPRYD7	SPRY Domain Containing 7	Other	Other	Protein-coding	7.94E−04	10
CUL3	Cullin 3	Enzyme	Nucleus	Protein-coding	7.97E−04	9
EPHA7	EPH Receptor A7	Kinase	Plasma membrane	Protein-coding	1.30E−03	10
TLK1	Tousled Like Kinase 1	Kinase	Nucleus	Protein-coding	7.94E−04	10
ICMT	Isoprenylcysteine carboxyl methyltransferase	Enzyme	Cytoplasm	Protein-coding	1.83E−03	11
GPD2	Glycerol-3-phosphate dehydrogenase 2	Enzyme	Cytoplasm	Protein-coding	2.26E−03	15
LRRC58	Leucine-Rich Repeat Containing 58	Other	Other	Protein-coding	1.49E−03	14

*FDR* false discovery rate

**Table 6:** Role of CDKN2B-AS1 in microRNA sponging in cancer. *Data source*: PubMed and NCBI

Cancer site	Sponged microRNA	Genes/pathway	Putative function	PubMed ID
**Breast (TNBC)**	miR-199a		Proliferation and apoptosis	28961506
Cervical cancer	miR-186		Cancer development	28550682
Oral cancer	miR-125a		Cell proliferation, migration, and invasion	29635126
Hepatocellular carcinoma	miR-122-5p		Cell proliferation, invasion, and metastasis	29127494
	miR-153-5p	ARHGAP18	Metastasis	30510148
	miR-384	STAT3		31679275
	miR-191	NF-κB and Wnt/β-catenin	Apoptosis, proliferation, metastasis, and invasion	30249208
	let-7c-5p	NAP1L1		30165194
	miR-122-5p		Cell proliferation, metastasis, and invasion	29127494
Gastric cancer	miR-99a	BMI1, Bcl-2	Cancer development	30156609
Colorectal	let-7a	ATP-binding cassette subfamily C member 1		30279206
Prostate cancer	let-7a	TGF-β1/Smad	Proliferation and migration	29278879
Glioma	miR-34a	Sirt1, PI3K/AKT, and mTOR	Cell proliferation, migration, and invasion	29057547
Retinoblastoma	miR-99a	c-MYC	Apoptosis, proliferation, metastasis and invasion	31184221
	miR-24	c-MYC, MEK/ERK, and Wnt/β-catenin	Viability, migration, and invasion	30703428
Osteosarcoma	miR-125a-5p	STAT3	Cisplatin chemoresistance	30777616
Nasopharyngeal carcinoma	miR-125a			28402230
	let-7a		Cisplatin chemoresistance	28117929
Medulloblastoma	miR-323	BRI3 and CDK6, p38 MAPK, ERK, and AKT, Wnt signaling	Cell proliferation and migration through miR-323-mediated regulation of BRI3	28513871

As one of the well-known lncRNAs that occur in genomic loci that harbor many cancer-associated SNPs [12], the *CDKN2B-AS1-*related variant rs2383207 showed, for the first time, an association with BC risk in the present study. The A/A homozygous carriers were three times more liable to develop BC under dominant and recessive genetic models. They were associated with short disease-free/overall survival compared to their counterpart G/G homozygous carriers. Intriguingly, the biallelic study variant was also associated with the gene expression, as G/G genotype was associated with a higher expression level than other genotypes (A/G and A/A).

Studies indicate that SNPs in *CDKN2B-AS1* can impact its expression and function with potential risk modification for diseases, including cancers [18, 52]. As most of the polymorphisms in this region do not impact any protein sequence, they likely act by affecting the expression of a nearby gene in cis, as proposed by Cunnington et al. [18]. Furthermore, as our in silico analysis showed, the existence of key genes highly enriched in BC with *CDKN2B-AS1* and upstream regulators enhancing the transcription of the *CDKN2B-AS1* gene (as detailed in Fig. 8) collectively suggests a wide "regulatory panorama" for this type of lncRNA [53]. Notably, it is not proved that the risky allele (the A allele in our case) is associated with high gene expression to increase BC susceptibility. As demonstrated in our in silico analysis and reported previously, the *CDKN2B-AS1* variants could act through "independent mechanisms" to increase/decrease susceptibility to diseases [53], and more than one functional variant as well as others in disequilibrium with the specified study variant, collectively, could impact *CDKN2B-AS1* expression [18].

In agreement with our results, the T allele of the rs2151280 variant (one of the *CDKN2B-AS1* SNPs) was related to increased susceptibility to neurofibromatosis type 1, associated with gene downregulation [54]. Also, the melanoma-associated rs1011970-T variant was reported to be associated with *CDKN2B-AS1* downregulation [18]. In contrast, the glioma-associated risky rs1063192-C allele was associated with increased *CDKN2B-AS1* expression [16, 18]. The presence of multiple regulatory elements and binding sites in the region of the studied variant suggests that its transcript, *CDKN2B-AS1*, with other genes in the same locus as *CDKN2A/B,* is subject to intricate "temporal and tissue-specific regulation" underlying the gene associations [53].

Based on the limited study cohort, future large-scale studies in other populations are warranted to confirm the studied variant’s association with BC risk. Also, supporting in vitro studies are needed to confirm the impact of different rs2383207 genotypes on *CDKN2B-AS1* expression. Finally, follow-up studies are warranted to assess the specified lncRNA’s validity as a diagnostic/prognostic epigenetic marker and the response to chemotherapy in BC patients.

## Conclusion

In summary, the study findings for the first time support the association of the *CDKN2B-AS1* rs2383207 (A>G) variant with gene expression and BC risk in the study population. It might provide new insight into the molecular stratification of BC patients. Also, high levels of circulating CDKN2B-AS1 could discriminate BC patients from controls and had prognostic value in patients’ survival, supporting its use in future epigenetic personalized BC treatment.

## Supplementary Information

**Additional file 1: Table S1.** Several types of long non-coding RNAs are involved in breast cancer. **Table S2.** Overlapping transcripts for rs2383207 variant according to NCBI and Varsome.com. **Table S3.** Role of CDKN2B-AS1 in cancer as a diagnostic and prognostic biomarker and therapeutic target.

## Data Availability

The data that support the findings of this study are available in the manuscript and the additional files.
