# Supplementary material for: Association of cyclin-dependent kinase inhibitor 2B antisense RNA 1 gene expression and rs2383207 variant with breast cancer risk and survival
Source: Cell Mol Biol Lett. 2021 Apr 13;26:14. doi: 10.1186/s11658-021-00258-9 (PMC8045214; doi:10.1186/s11658-021-00258-9)
Supplement: Supplementary file 1 — Additional file 1: Table S1. Several types of long non-coding RNAs are involved in breast cancer. Table S2. Overlapping transcripts for rs2383207 variant according to NCBI and Varsome.com. Table S3. Role of CDKN2B-AS1 in cancer as a diagnostic and prognostic biomarker and therapeutic target. [file 11658_2021_258_MOESM1_ESM.docx]

**Table S1 Several types of long non-coding RNAs are involved in breast cancer**

| **# lncRNA** | **LncRNA name** | **Cancer name** | **Expression pattern** | **Drug-resistant** | **Circulating** | **Prognostic** | **PubMed ID** |
| --- | --- | --- | --- | --- | --- | --- | --- |
| 1 | 7SL | BC | differential expression | ✘ | ✘ | ✘ | 26718402 |
| 2 | 91H | BC | up-regulated | ✘ | ✘ | ✘ | 27780718 |
| 3 | AC026904.1 | BC | upregulated | ✘ | ✘ | ✔ | 29774079 |
| 4 | AF251187 | BC | upregulated | ✔ | ✔ | ✔ | 28003470 |
| 5 | AFAP1-AS1 | BC | upregulated | ✘ | ✘ | ✘ | 29439313 |
| 6 | AFAP1-AS1 | BC | upregulated | ✘ | ✘ | ✔ | 29974352 |
| 7 | AK001796 | BC | upregulated | ✘ | ✘ | ✔ | 27897214 |
| 8 | AK024898 | BC | upregulated | ✘ | ✘ | ✘ | 28003470 |
| 9 | AK025743 | BC | upregulated | ✘ | ✘ | ✘ | 28003470 |
| 10 | AK057709 | BC | upregulated | ✘ | ✘ | ✘ | 28003470 |
| 11 | AK090603 | BC | upregulated | ✘ | ✘ | ✘ | 28003470 |
| 12 | AK096780 | BC | downregulated | ✘ | ✘ | ✘ | 28003470 |
| 13 | AK123408 | BC | downregulated | ✔ | ✔ | ✔ | 28003470 |
| 14 | AK124454 | BC | upregulated | ✘ | ✘ | ✔ | 26921339 |
| 15 | AK127565 | BC | upregulated | ✔ | ✔ | ✔ | 28003470 |
| 16 | AL832444 | BC | downregulated | ✔ | ✔ | ✔ | 28003470 |
| 17 | AL833160 | BC | upregulated | ✔ | ✔ | ✔ | 28003470 |
| 18 | ANAC | BC | downregulated | ✘ | ✘ | ✘ | 28978036 |
| 19 | ANRASSF1 | BC | upregulated | ✔ | ✘ | ✔ | 23990798 |
|  | ANRASSF1 | BC | upregulated | ✘ | ✘ | ✘ | 26409453 |
| 20 | CDKN2B-AS1 | BC | Upregulated | ✘ | ✘ | ✘ | 31572679 |
|  | CDKN2B-AS1 | TNBC | upregulated | ✘ | ✘ | ✔ | 28961506 |
|  | CDKN2B-AS1 | BC | upregulated | ✘ | ✔ | ✔ | 28248879 |
|  | CDKN2B-AS1 | BC | upregulated | ✘ | ✘ | ✘ | 26835415 |
|  | CDKN2B-AS1 | BC | upregulated | ✘ | ✘ | ✘ | 17440112 |
|  | CDKN2B-AS1 | BC | upregulated | ✘ | ✘ | ✘ | 26409453 |
| 21 | AP000439.3 | BC | upregulated | ✘ | ✘ | ✘ | 29048636 |
| 22 | ARNILA | TNBC | upregulated | ✘ | ✘ | ✔ | 29844570 |
| 23 | ASBEL | BC | downregulated | ✘ | ✔ | ✔ | 28552529 |
| 24 | ASHGA5P014632 | BC | upregulated | ✘ | ✘ | ✔ | 27322459 |
| 25 | ASHGA5P018902 | TNBC | upregulated | ✘ | ✘ | ✘ | 26910840 |
| 26 | AX747207 | BC | downregulated | ✘ | ✘ | ✘ | 29749447 |
| 27 | BANCR | BC | upregulated | ✘ | ✘ | ✔ | 29565494 |
|  | BANCR | BC | upregulated | ✘ | ✘ | ✔ | 29805676 |
| 28 | BC016787 | BC | upregulated | ✔ | ✔ | ✔ | 28003470 |
| 29 | BC016831 | TNBC | downregulated | ✘ | ✘ | ✘ | 26910840 |
| 30 | BC036599 | BC | downregulated | ✔ | ✔ | ✔ | 28003470 |
| 31 | BC038366 | BC | upregulated | ✔ | ✔ | ✔ | 28003470 |
| 32 | BC038580 | BC | upregulated | ✔ | ✔ | ✔ | 28003470 |
| 33 | BC039678 | BC | upregulated | ✔ | ✔ | ✔ | 28003470 |
| 34 | BC040572 | BC | upregulated | ✔ | ✔ | ✔ | 28003470 |
| 35 | BC040587 | BC | downregulated | ✘ | ✘ | ✔ | 25435812 |
| 36 | BC041455 | BC | downregulated | ✔ | ✔ | ✔ | 28003470 |
| 37 | BC200 | BC | upregulated | ✘ | ✘ | ✔ | 28651607 |
|  | BC200 | BC | upregulated | ✔ | ✘ | ✔ | 27277684 |
|  | BC200 | BC | upregulated | ✘ | ✘ | ✘ | 15240511 |
|  | BC200 | BC | upregulated | ✘ | ✘ | ✘ | 9422992 |
| 38 | BCAR4 | BC | differential expression | ✔ | ✘ | ✔ | 22892392 |
|  | BCAR4 | BC | upregulated | ✔ | ✘ | ✔ | 21506106 |
|  | BCAR4 | BC | upregulated | ✔ | ✘ | ✔ | 20859285 |
|  | BCAR4 | BC | upregulated | ✔ | ✘ | ✔ | 16778085 |
|  | BCAR4 | BC | upregulated | ✘ | ✘ | ✔ | 26550436 |
|  | BCAR4 | BC | upregulated | ✔ | ✘ | ✔ | 26317614 |
| 39 | BORG | BC | upregulated | ✘ | ✔ | ✔ | 28983112 |
| 40 | CASC2 | BC | downregulated | ✘ | ✘ | ✘ | 29523222 |
| 41 | CASIMO1 | BC | upregulated | ✘ | ✘ | ✘ | 29765154 |
| 42 | CCAT1 | BC | upregulated | ✘ | ✘ | ✔ | 26464701 |
|  | CCAT1 | BC | upregulated | ✘ | ✘ | ✔ | 29024383 |
| 43 | CCAT2 | BC | downregulated | ✘ | ✘ | ✔ | 28480695 |
|  | CCAT2 | BC | upregulated | ✘ | ✘ | ✘ | 28272713 |
|  | CCAT2 | BC | upregulated | ✘ | ✘ | ✔ | 28531944 |
|  | CCAT2 | BC | upregulated | ✘ | ✔ | ✔ | 26442763 |
|  | CCAT2 | BC | upregulated | ✔ | ✘ | ✘ | 24077681 |
| 44 | CPSF6 | BC | upregulated | ✘ | ✘ | ✔ | 28673861 |
| 45 | CR592608 | BC | upregulated | ✘ | ✘ | ✘ | 28003470 |
| 46 | CR593775 | BC | upregulated | ✘ | ✘ | ✘ | 28003470 |
| 47 | CR610499 | BC | downregulated | ✘ | ✘ | ✘ | 28003470 |
| 48 | CR612213 | BC | upregulated | ✘ | ✘ | ✘ | 28003470 |
| 49 | CRALA | BC | upregulated | ✔ | ✘ | ✔ | 28834648 |
| 50 | CRNDE | BC | upregulated | ✘ | ✘ | ✔ | 28469804 |
| 51 | CTC-338M12.3 | TNBC | upregulated | ✘ | ✘ | ✘ | 26910840 |
| 52 | DANCR | BC | downregulated | ✘ | ✘ | ✘ | 27716745 |
|  | DANCR | TNBC | upregulated | ✘ | ✘ | ✔ | 28760736 |
| 53 | DSCAM-AS1 | BC | upregulated | ✔ | ✘ | ✘ | 29462945 |
|  | DSCAM-AS1 | BC | upregulated | ✘ | ✘ | ✘ | 26621851 |
|  | DSCAM-AS1 | BC | upregulated | ✔ | ✘ | ✘ | 27666543 |
|  | DSCAM-AS1 | BC | upregulated | ✘ | ✘ | ✘ | 12177779 |
| 54 | DUXAP10 | BC | upregulated | ✘ | ✘ | ✔ | 27322459 |
| 55 | EFNA3 | BC | upregulated | ✘ | ✘ | ✔ | 25023702 |
| 56 | EGOT | BC | downregulated | ✘ | ✘ | ✔ | 26159853 |
| 57 | EPB41L4A-AS2 | BC | downregulated | ✘ | ✘ | ✔ | 26980733 |
| 58 | EPIC1 | BC | upregulated | ✘ | ✘ | ✔ | 29622465 |
| 59 | ESRPS-AS | BC | upregulated | ✘ | ✘ | ✔ | 28759043 |
| 60 | FAL1 | BC | upregulated | ✘ | ✘ | ✔ | 25203321 |
| 61 | FENDRR | BC | downregulated | ✘ | ✘ | ✔ | 29559798 |
| 62 | FEZF1-AS1 | BC | upregulated | ✘ | ✘ | ✘ | 29797562 |
| 63 | FGF14-AS2 | BC | downregulated | ✘ | ✘ | ✔ | 26820525 |
| 64 | FOXC2-AS1 | BC | upregulated | ✘ | ✘ | ✔ | 29562954 |
| 65 | FOXCUT | TNBC | upregulated | ✘ | ✘ | ✘ | 25516208 |
| 66 | FTH1P3 | BC | upregulated | ✔ | ✘ | ✔ | 29971911 |
| 67 | GACAT3 | BC | upregulated | ✘ | ✘ | ✘ | 29945347 |
| 68 | GAS5 | BC | downregulated | ✘ | ✘ | ✘ | 23933812 |
|  | GAS5 | BC | downregulated | ✔ | ✔ | ✘ | 29655698 |
|  | GAS5 | BC | downregulated | ✘ | ✘ | ✘ | 27922078 |
|  | GAS5 | BC | downregulated | ✘ | ✔ | ✘ | 26662314 |
|  | GAS5 | BC | downregulated | ✘ | ✘ | ✔ | 24789445 |
|  | GAS5 | BC | downregulated | ✘ | ✘ | ✔ | 18836484 |
|  | GAS5 | BC | downregulated | ✔ | ✘ | ✔ | 27034004 |
|  | GAS5 | BC | downregulated | ✘ | ✘ | ✔ | 26550436 |
|  | GAS5 | BC | differential expression | ✘ | ✘ | ✘ | 29785740 |
|  | GAS5 | BC | downregulated | ✘ | ✘ | ✔ | 29793177 |
|  | GAS5 | BC | downregulated | ✘ | ✘ | ✘ | 30007957 |
|  | GAS5 | BC | down-regulation | ✔ | ✘ | ✔ | 29969658 |
| 69 | GHET1 | BC | upregulated | ✘ | ✘ | ✔ | 29843220 |
| 70 | GNG12-AS1 | BC | downregulated | ✘ | ✘ | ✘ | 23871723 |
| 71 | H19 | BC | upregulated | ✘ | ✘ | ✔ | 29693231 |
|  | H19 | BC | upregulated | ✔ | ✘ | ✔ | 29106390 |
|  | H19 | BC | upregulated | ✘ | ✘ | ✘ | 26353930 |
|  | H19 | BC | upregulated | ✘ | ✘ | ✘ | 28611183 |
|  | H19 | BC | downregulated | ✘ | ✘ | ✘ | 29737472 |
|  | H19 | BC | differential expression | ✔ | ✘ | ✘ | 29145193 |
|  | H19 | BC | upregulated | ✔ | ✘ | ✔ | 29190892 |
|  | H19 | BC | upregulated | ✘ | ✘ | ✘ | 28919786 |
|  | H19 | BC | upregulated | ✘ | ✔ | ✘ | 27540977 |
|  | H19 | BC | upregulated | ✔ | ✘ | ✔ | 27845892 |
|  | H19 | BC | upregulated | ✘ | ✘ | ✘ | 26323944 |
|  | H19 | BC | differential expression | ✘ | ✘ | ✘ | 25944846 |
|  | H19 | BC | differential expression | ✘ | ✘ | ✔ | 21748294 |
|  | H19 | BC | upregulated | ✘ | ✘ | ✘ | 16707459 |
|  | H19 | BC | differential expression | ✘ | ✘ | ✘ | 14729626 |
|  | H19 | BC | upregulated | ✘ | ✘ | ✘ | 11896592 |
|  | H19 | BC | upregulated | ✘ | ✘ | ✘ | 12419837 |
|  | H19 | BC | upregulated | ✘ | ✘ | ✘ | 9811352 |
|  | H19 | BC | upregulated | ✘ | ✘ | ✘ | 9811352 |
|  | H19 | BC | differential expression | ✘ | ✘ | ✘ | 8636375 |
|  | H19 | BC | upregulated | ✔ | ✘ | ✘ | 8674037 |
|  | H19 | BC | upregulated | ✘ | ✘ | ✘ | 8785513 |
|  | H19 | BC | upregulated | ✘ | ✘ | ✔ | 28102845 |
|  | H19 | BC | upregulated | ✘ | ✘ | ✘ | 27780718 |
|  | H19 | BC | upregulated | ✘ | ✘ | ✘ | 28544374 |
|  | H19 | BC | upregulated | ✘ | ✘ | ✔ | 25846769 |
|  | H19 | BC | upregulated | ✘ | ✘ | ✘ | 18794369 |
|  | H19 | BC | upregulated | ✘ | ✘ | ✘ | 15985428 |
|  | H19 | BC | down-regulation | ✔ | ✘ | ✔ | 29963109 |
| 72 | HIF1A-AS2 | BC | upregulated | ✘ | ✔ | ✘ | 28248879 |
|  | HIF1A-AS2 | BC | upregulated | ✘ | ✘ | ✔ | 14580258 |
|  | HIF1A-AS2 | BC | upregulated | ✘ | ✔ | ✘ | 26921339 |
| 73 | HMMR-AS1 | BC | upregulated | ✘ | ✘ | ✘ | 27920576 |
| 74 | HOTAIR | BC | upregulated | ✘ | ✔ | ✘ | 29687854 |
|  | HOTAIR | BC | differential expression | ✘ | ✔ | ✘ | 25586347 |
|  | HOTAIR | BC | upregulated | ✔ | ✘ | ✘ | 25883211 |
|  | HOTAIR | BC | upregulated | ✘ | ✔ | ✘ | 26033707 |
|  | HOTAIR | TNBC | upregulated | ✘ | ✘ | ✘ | 25996380 |
|  | HOTAIR | BC | upregulated | ✔ | ✔ | ✘ | 25928008 |
|  | HOTAIR | BC | upregulated | ✘ | ✘ | ✘ | 25070049 |
|  | HOTAIR | BC | upregulated | ✘ | ✘ | ✘ | 29325547 |
|  | HOTAIR | BC | differential expression | ✘ | ✘ | ✘ | 28069441 |
|  | HOTAIR | BC | upregulated | ✘ | ✘ | ✘ | 28122024 |
|  | HOTAIR | BC | upregulated | ✘ | ✘ | ✘ | 28454226 |
|  | HOTAIR | BC | differential expression | ✔ | ✘ | ✔ | 28420874 |
|  | HOTAIR | BC | differential expression | ✘ | ✘ | ✘ | 28407576 |
|  | HOTAIR | BC | upregulated | ✘ | ✔ | ✘ | 27755794 |
|  | HOTAIR | BC | upregulated | ✔ | ✘ | ✔ | 27378691 |
|  | HOTAIR | BC | upregulated | ✘ | ✘ | ✘ | 26323944 |
|  | HOTAIR | BC | downregulated | ✔ | ✘ | ✘ | 25613518 |
|  | HOTAIR | BC | upregulated | ✘ | ✘ | ✘ | 24533973 |
|  | HOTAIR | BC | upregulated | ✘ | ✘ | ✘ | 25328122 |
|  | HOTAIR | BC | upregulated | ✔ | ✘ | ✘ | 23375982 |
|  | HOTAIR | BC | upregulated | ✘ | ✘ | ✘ | 24022994 |
|  | HOTAIR | BC | differential expression | ✘ | ✘ | ✔ | 23124417 |
|  | HOTAIR | BC | upregulated | ✘ | ✘ | ✔ | 23133536 |
|  | HOTAIR | BC | upregulated | ✘ | ✘ | ✔ | 20393566 |
|  | HOTAIR | BC | upregulated | ✔ | ✘ | ✘ | 27388461 |
|  | HOTAIR | BC | upregulated | ✘ | ✘ | ✘ | 28846832 |
|  | HOTAIR | BC | upregulated | ✘ | ✘ | ✔ | 29473328 |
|  | HOTAIR | BC | upregulated | ✘ | ✘ | ✔ | 29222472 |
|  | HOTAIR | BC | upregulated | ✘ | ✘ | ✔ | 29229759 |
|  | HOTAIR | BC | upregulated | ✔ | ✘ | ✘ | 26364613 |
|  | HOTAIR | BC | upregulated | ✘ | ✘ | ✔ | 26550436 |
| 75 | HOTTIP | BC | upregulated | ✔ | ✘ | ✘ | 29415429 |
| 76 | HOXA11-AS | BC | upregulated | ✘ | ✘ | ✘ | 28791375 |
|  | HOXA11-AS | BC | upregulated | ✘ | ✘ | ✘ | 28701685 |
| 77 | HOXA-AS2 | BC | upregulated | ✘ | ✘ | ✘ | 28545023 |
| 78 | HTF30525 | BC | upregulated | ✘ | ✘ | ✘ | 28003470 |
| 79 | HULC | BC | upregulated | ✘ | ✘ | ✔ | 27986124 |
| 80 | IGFL2-AS1 | BC | downregulated | ✘ | ✘ | ✘ | 28488769 |
| 81 | IGKV | TNBC | downregulated | ✘ | ✘ | ✘ | 26910840 |
| 82 | INXS | BC | downregulated | ✘ | ✘ | ✘ | 24992962 |
| 83 | IRAIN | BC | downregulated | ✘ | ✔ | ✘ | 25465188 |
| 84 | ITGB2-AS1 | BC | downregulated | ✘ | ✘ | ✔ | 29941860 |
| 85 | KCNQ1OT1 | BC | upregulated | ✘ | ✘ | ✘ | 26323944 |
| 86 | LINC00052 | BC | upregulated | ✘ | ✘ | ✘ | 28036286 |
|  | LINC00052 | TNBC | downregulated | ✘ | ✘ | ✘ | 26910840 |
|  | LINC00152 | TNBC | upregulated | ✘ | ✘ | ✘ | 29156515 |
|  | LINC00152 | BC | differential expression | ✘ | ✘ | ✘ | 29268251 |
|  | LINC00152 | BC | upregulated | ✔ | ✘ | ✘ | 29863253 |
| 87 | LINC00310 | BC | upregulated | ✘ | ✔ | ✔ | 29993199 |
| 88 | LINC00472 | BC | downregulated | ✘ | ✘ | ✔ | 25865225 |
| 89 | Linc00518 | BC | upregulated | ✔ | ✘ | ✘ | 30001527 |
| 90 | LINC00520 | BC | upregulated | ✘ | ✘ | ✘ | 27626181 |
| 91 | LINC00628 | BC | downregulated | ✘ | ✘ | ✔ | 28165561 |
| 92 | LINC00663 | BC | downregulated | ✘ | ✘ | ✘ | 26743782 |
| 93 | LINC00673 | BC | upregulated | ✘ | ✘ | ✔ | 28795861 |
| 94 | LINC00704 | BC | upregulated | ✔ | ✘ | ✔ | 29378907 |
| 95 | LINC00894-002 | BC | downregulated | ✔ | ✘ | ✘ | 29738694 |
| 96 | LINC01089 | BC | downregulated | ✘ | ✘ | ✔ | 27485121 |
| 97 | LINC01296 | BC | upregulated | ✘ | ✘ | ✔ | 29981416 |
| 98 | linc01561 | BC | downregulated | ✘ | ✘ | ✘ | 29890225 |
| 99 | LINC01638 | BC | upregulated | ✘ | ✘ | ✔ | 30002443 |
| 100 | linc-GGTLC1-7 | BC | downregulated | ✘ | ✘ | ✘ | 25749757 |
| 101 | LincIN | BC | upregulated | ✘ | ✘ | ✘ | 28558830 |
| 102 | linc-ITGB1 | BC | downregulated | ✘ | ✘ | ✔ | 28829502 |
|  | linc-ITGB1 | BC | upregulated | ✘ | ✘ | ✘ | 26601916 |
| 103 | linc-POU3F3 | BC | up-regulated | ✘ | ✘ | ✘ | 26550436 |
| 104 | linc-PSMD-11 | BC | up-regulated | ✘ | ✘ | ✘ | 25749757 |
| 105 | lincRNA-APOC1P1-3 | BC | up-regulated | ✘ | ✘ | ✘ | 27228351 |
| 106 | lincRNA-BC2 | BC | up-regulated | ✘ | ✘ | ✘ | 25084155 |
| 107 | lincRNA-BC4 | BC | downregulated | ✘ | ✘ | ✘ | 25084155 |
| 108 | lincRNA-BC5 | BC | up-regulated | ✘ | ✘ | ✘ | 25084155 |
| 109 | lincRNA-BC8 | BC | downregulated | ✘ | ✘ | ✘ | 25084155 |
| 110 | lincRNA-p21 | BC | up-regulated | ✘ | ✘ | ✘ | 26656491 |
| 111 | linc-ROR | TNBC | downregulated | ✘ | ✘ | ✘ | 29673594 |
|  | linc-ROR | BC | upregulated | ✘ | ✘ | ✘ | 27449099 |
|  | linc-ROR | BC | upregulated | ✘ | ✘ | ✘ | 24922071 |
|  | linc-ROR | BC | upregulated | ✔ | ✘ | ✘ | 28063065 |
|  | linc-ROR | BC | downregulated | ✘ | ✘ | ✘ | 26314857 |
|  | linc-ROR | BC | upregulated | ✘ | ✘ | ✘ | 23208419 |
|  | linc-ROR | BC | upregulated | ✔ | ✘ | ✔ | 29041978 |
|  | linc-ROR | BC | upregulated | ✘ | ✘ | ✘ | 25253741 |
|  | linc-ROR | BC | upregulated | ✔ | ✔ | ✔ | 28869448 |
|  | linc-ROR | BC | upregulated | ✔ | ✘ | ✘ | 26883251 |
|  | linc-ROR | BC | upregulated | ✘ | ✔ | ✔ | 29090518 |
|  | linc-ROR | BC | differential expression | ✔ | ✘ | ✘ | 28635401 |
| 112 | linc-TPBG-3 | BC | up-regulated | ✘ | ✘ | ✘ | 25749757 |
| 113 | linc-TRIP11 | BC | up-regulated | ✘ | ✘ | ✘ | 25749757 |
| 114 | LINK-A | BC | differential expression | ✘ | ✘ | ✔ | 28218907 |
| 115 | LINP1 | BC | upregulated | ✔ | ✘ | ✔ | 29293402 |
|  | LINP1 | BC | upregulated | ✘ | ✘ | ✘ | 27111890 |
| 116 | LncCAMTA1 | BC | differential expression | ✘ | ✘ | ✘ | 28550685 |
| 117 | lncFOXO1 | BC | downregulated | ✘ | ✘ | ✔ | 28339037 |
| 118 | lnc-HOST2 | BC | upregulated | ✘ | ✘ | ✘ | 29236319 |
| 119 | lncRNA00544 | BC | up-regulated | ✘ | ✔ | ✔ | 28959047 |
| 120 | lncRNAâ-MIF | BC | differential expression | ✘ | ✘ | ✘ | 27317567 |
| 121 | lncRNA-AK058803 | BC | up-regulated | ✘ | ✘ | ✘ | 26136884 |
| 122 | lncRNA-ATB | BC | up-regulated | ✔ | ✘ | ✘ | 25871474 |
|  | lncRNA-ATB | BC | downregulated | ✘ | ✘ | ✘ | 28598827 |
| 123 | lncRNA-BCHE | BC | up-regulated | ✘ | ✘ | ✔ | 29948648 |
| 124 | lncRNA-CTD-2108O9.1 | BC | downregulated | ✔ | ✘ | ✘ | 29603493 |
| 125 | lncRNA-Hh | BC | upregulated | ✘ | ✘ | ✘ | 26418365 |
| 126 | lncRNA-HIT | BC | up-regulated | ✘ | ✘ | ✘ | 25605728 |
| 127 | lncRNA-JADE | BC | up-regulated | ✘ | ✘ | ✘ | 24097061 |
| 128 | lncRNA-PRLB | BC |  | ✔ | ✔ | ✔ | 29752439 |
| 129 | LOC284454 | BC | downregulated | ✘ | ✘ | ✘ | 29227193 |
| 130 | LOC441242 | TNBC | downregulated | ✘ | ✘ | ✘ | 26910840 |
| 131 | LOC554202 | BC | downregulated | ✘ | ✘ | ✘ | 22289355 |
|  | LOC554202 | BC | upregulated | ✘ | ✘ | ✘ | 24631686 |
| 132 | LSINCT1 | BC | upregulated | ✘ | ✘ | ✘ | 20214974 |
| 133 | LSINCT10 | BC | upregulated | ✘ | ✘ | ✘ | 20214974 |
| 134 | LSINCT11 | BC | upregulated | ✘ | ✘ | ✘ | 20214974 |
| 135 | LSINCT12 | BC | upregulated | ✘ | ✘ | ✘ | 20214974 |
| 136 | LSINCT2 | BC | upregulated | ✘ | ✘ | ✘ | 20214974 |
| 137 | LSINCT3 | BC | upregulated | ✘ | ✘ | ✘ | 20214974 |
| 138 | LSINCT4 | BC | upregulated | ✘ | ✘ | ✘ | 20214974 |
| 139 | LSINCT5 | BC | upregulated | ✘ | ✘ | ✘ | 21532345 |
| 140 | LSINCT5 | BC | upregulated | ✘ | ✘ | ✘ | 20214974 |
| 141 | LSINCT5 | BC | differential expression | ✘ | ✘ | ✘ | 29785740 |
| 142 | LSINCT6 | BC | upregulated | ✘ | ✘ | ✘ | 20214974 |
| 143 | LSINCT7 | BC | upregulated | ✘ | ✘ | ✘ | 20214974 |
| 144 | LSINCT8 | BC | upregulated | ✘ | ✘ | ✘ | 20214974 |
| 145 | LSINCT9 | BC | upregulated | ✘ | ✘ | ✘ | 20214974 |
| 146 | LUNAR1 | BC | downregulated | ✘ | ✘ | ✘ | 26550436 |
| 147 | MAGI2-AS3 | BC | downregulated | ✘ | ✘ | ✘ | 29679339 |
| 148 | MALAT1 | BC | upregulated | ✘ | ✔ | ✘ | 29310836 |
|  | MALAT1 | TNBC | upregulated | ✘ | ✘ | ✘ | 25996380 |
|  | MALAT1 | BC | upregulated | ✘ | ✘ | ✘ | 29130936 |
|  | MALAT1 | BC | downregulated | ✘ | ✘ | ✔ | 29574704 |
|  | MALAT1 | BC | upregulated | ✘ | ✘ | ✘ | 26926567 |
|  | MALAT1 | BC | downregulated | ✘ | ✘ | ✘ | 26275461 |
|  | MALAT1 | BC | upregulated | ✘ | ✘ | ✔ | 26676637 |
|  | MALAT1 | BC | upregulated | ✘ | ✔ | ✘ | 29683112 |
|  | MALAT1 | BC | upregulated | ✘ | ✔ | ✘ | 29146194 |
|  | MALAT1 | BC | differential expression | ✘ | ✘ | ✔ | 28652379 |
|  | MALAT1 | BC | upregulated | ✘ | ✘ | ✘ | 29434914 |
|  | MALAT1 | BC | upregulated | ✘ | ✘ | ✘ | 27172249 |
|  | MALAT1 | BC | differential expression | ✘ | ✘ | ✘ | 27197265 |
|  | MALAT1 | BC | upregulated | ✘ | ✔ | ✔ | 27250026 |
|  | MALAT1 | BC | upregulated | ✘ | ✘ | ✘ | 27514584 |
|  | MALAT1 | BC | upregulated | ✘ | ✘ | ✘ | 24525122 |
|  | MALAT1 | BC | upregulated | ✘ | ✘ | ✔ | 28675122 |
|  | MALAT1 | BC | upregulated | ✘ | ✘ | ✘ | 26918449 |
|  | MALAT1 | BC | upregulated | ✘ | ✘ | ✘ | 26917489 |
|  | MALAT1 | BC | upregulated | ✘ | ✘ | ✔ | 29416769 |
|  | MALAT1 | BC | upregulated | ✘ | ✘ | ✔ | 28915533 |
|  | MALAT1 | BC | upregulated | ✘ | ✔ | ✔ | 27466303 |
|  | MALAT1 | BC | upregulated | ✘ | ✘ | ✔ | 27191888 |
|  | MALAT1 | BC | downregulated | ✘ | ✘ | ✔ | 26191181 |
|  | MALAT1 | BC | upregulated | ✘ | ✘ | ✘ | 26550436 |
|  | MALAT1 | BC | upregulated | ✘ | ✘ | ✔ | 29912916 |
| 149 | MAPT-AS1 | BC | upregulated | ✘ | ✘ | ✘ | 29441192 |
| 150 | MEG3 | BC | downregulated | ✘ | ✘ | ✘ | 28635399 |
|  | MEG3 | BC | downregulated | ✘ | ✘ | ✘ | 27166155 |
|  | MEG3 | BC | downregulated | ✘ | ✘ | ✔ | 28051255 |
|  | MEG3 | BC | downregulated | ✔ | ✘ | ✔ | 28463794 |
|  | MEG3 | BC | downregulated | ✘ | ✘ | ✘ | 14602737 |
| 151 | MIAT | BC | upregulated | ✘ | ✘ | ✘ | 29100300 |
|  | MIAT | BC | upregulated | ✘ | ✘ | ✘ | 29345338 |
|  | MIAT | BC | upregulated | ✘ | ✘ | ✘ | 29792859 |
| 152 | MVIH | BC | upregulated | ✘ | ✘ | ✔ | 26555546 |
| 153 | MYCLo-5 | BC | downregulated | ✘ | ✘ | ✘ | 26003165 |
|  | MYCLo-5 | BC | downregulated | ✘ | ✘ | ✘ | 26003165 |
| 154 | NBAT1 | BC | downregulated | ✘ | ✘ | ✔ | 26378045 |
| 155 | NCRMS | BC | downregulated | ✘ | ✘ | ✘ | 29215701 |
| 156 | NEAT1 | BC | upregulated | ✘ | ✘ | ✘ | 29323713 |
|  | NEAT1 | BC | downregulated | ✘ | ✘ | ✘ | 27147820 |
|  | NEAT1 | BC | upregulated | ✘ | ✘ | ✘ | 27556296 |
|  | NEAT1 | BC | upregulated | ✘ | ✘ | ✘ | 27514584 |
|  | NEAT1 | BC | upregulated | ✘ | ✘ | ✔ | 25417700 |
|  | NEAT1 | BC | upregulated | ✔ | ✘ | ✔ | 28720546 |
|  | NEAT1 | BC | upregulated | ✘ | ✘ | ✘ | 28034643 |
|  | NEAT1 | BC | downregulated | ✘ | ✘ | ✔ | 28946559 |
|  | NEAT1 | BC | upregulated | ✘ | ✘ | ✔ | 28338194 |
| 157 | NKILA | BC | upregulated | ✘ | ✘ | ✔ | 29761481 |
|  | NKILA | BC | downregulated | ✘ | ✘ | ✔ | 25759022 |
| 158 | NNT-AS1 | BC | upregulated | ✘ | ✘ | ✔ | 29710510 |
| 159 | NONHSAT012762 | TNBC | upregulated | ✘ | ✘ | ✘ | 26078338 |
| 160 | NONHSAT125629 | TNBC | downregulated | ✘ | ✘ | ✘ | 26078338 |
| 161 | NORAD | BC | upregulated | ✘ | ✘ | ✔ | 26942882 |
| 162 | OR3A4 | BC | upregulated | ✘ | ✘ | ✔ | 29031201 |
| 163 | PANDAR | BC | upregulated | ✘ | ✘ | ✘ | 26927017 |
| 164 | PARTICLE | BC | differential expression | ✘ | ✔ | ✔ | 28769061 |
| 165 | PAX8-AS1-N | BC | downregulated | ✔ | ✘ | ✔ | 29693272 |
| 166 | PCAN-1 | BC | upregulated | ✘ | ✘ | ✔ | 27322459 |
| 167 | PCAN-4 | BC | downregulated | ✘ | ✘ | ✔ | 27322459 |
| 168 | PCAN-6 | BC | upregulated | ✘ | ✘ | ✔ | 27322459 |
| 169 | PCAT-1 | BC | upregulated | ✘ | ✘ | ✘ | 28989584 |
| 170 | PCAT6 | TNBC | upregulated | ✘ | ✘ | ✘ | 25996380 |
| 171 | PICART1 | BC | downregulated | ✘ | ✘ | ✘ | 28339031 |
| 172 | PlncRNA-1 | BC | downregulated | ✘ | ✔ | ✘ | 29626321 |
| 173 | PTENP1 | BC | downregulated | ✔ | ✘ | ✘ | 29085464 |
|  | PTENP1 | BC | downregulated | ✘ | ✘ | ✔ | 29212574 |
| 174 | PTPRG-AS1 | BC | upregulated | ✘ | ✘ | ✘ | 26409453 |
| 175 | PVT1 | BC | downregulated | ✘ | ✘ | ✔ | 28480695 |
|  | PVT1 | BC | upregulated | ✘ | ✘ | ✔ | 29760406 |
|  | PVT1 | BC | upregulated | ✘ | ✘ | ✘ | 29731168 |
|  | PVT1 | BC | upregulated | ✘ | ✘ | ✔ | 28882595 |
|  | PVT1 | BC | upregulated | ✘ | ✘ | ✔ | 17908964 |
|  | PVT1 | BC | downregulated | ✘ | ✔ | ✔ | 28534994 |
|  | PVT1 | BC | upregulated | ✘ | ✘ | ✘ | 26550436 |
|  | PVT1 | BC | upregulated | ✘ | ✘ | ✘ | 24780616 |
| 176 | RP11-434D9.1 | TNBC | downregulated | ✘ | ✘ | ✘ | 26910840 |
| 177 | RP11-445H22.4 | BC | upregulated | ✘ | ✔ | ✘ | 25929808 |
| 178 | RP4-583P15.10 | BC | upregulated | ✘ | ✘ | ✘ | 25661361 |
| 179 | RP4-781K5.4 | TNBC | downregulated | ✘ | ✘ | ✘ | 26910840 |
| 180 | RPPH1 | BC | upregulated | ✘ | ✘ | ✘ | 29200969 |
| 181 | SKAI1BC | TNBC | upregulated | ✘ | ✘ | ✘ | 28978052 |
| 182 | snaR | BC | up-regulated | ✘ | ✘ | ✘ | 27919948 |
| 183 | SNHG12 | BC | upregulated | ✘ | ✘ | ✔ | 28337281 |
| 184 | SNHG14 | BC | upregulated | ✔ | ✘ | ✘ | 30015837 |
| 185 | SNHG15 | BC | upregulated | ✘ | ✘ | ✔ | 29217194 |
|  | SNHG15 | BC | downregulated | ✘ | ✘ | ✘ | 29886172 |
| 186 | SNHG16 | BC | upregulated | ✘ | ✘ | ✘ | 28232182 |
| 187 | SOX2 | BC | upregulated | ✘ | ✘ | ✔ | 28882595 |
| 188 | SOX2OT | BC | upregulated | ✘ | ✔ | ✘ | 28240100 |
|  | SOX2OT | BC | upregulated | ✘ | ✘ | ✘ | 26703382 |
|  | SOX2OT | BC | upregulated | ✘ | ✘ | ✘ | 25006803 |
|  | SOX2OT | BC | upregulated | ✘ | ✘ | ✘ | 26409453 |
| 189 | SPRY4-IT1 | BC | upregulated | ✘ | ✘ | ✘ | 25742952 |
| 190 | SRA1 | BC | differential expression | ✘ | ✘ | ✘ | 10485452 |
|  | SRA1 | BC | differential expression | ✘ | ✘ | ✘ | 20079837 |
|  | SRA1 | BC | differential expression | ✘ | ✘ | ✘ | 16848684 |
|  | SRA1 | BC | upregulated | ✘ | ✘ | ✘ | 19483093 |
| 191 | SUMO1P3 | BC | upregulated | ✔ | ✘ | ✔ | 29312511 |
| 192 | TCONS_l2_00003938 | TNBC | upregulated | ✘ | ✘ | ✘ | 25996380 |
| 193 | TINCR | BC | upregulated | ✘ | ✔ | ✔ | 29614984 |
| 194 | TP73-AS1 | BC | upregulated | ✔ | ✘ | ✔ | 28639399 |
|  | TP73-AS1 | BC | downregulated | ✘ | ✘ | ✘ | 28857253 |
| 195 | TRERNA1 | BC | upregulated | ✘ | ✘ | ✘ | 23974796 |
| 196 | TUG1 | BC | downregulated | ✘ | ✘ | ✔ | 28950664 |
|  | TUG1 | BC | upregulated | ✘ | ✘ | ✘ | 28053623 |
|  | TUG1 | BC | upregulated | ✘ | ✘ | ✘ | 27848085 |
| 197 | TUNAR | BC | upregulated | ✘ | ✘ | ✘ | 26207516 |
| 198 | TUSC7 | BC | differential expression | ✘ | ✘ | ✘ | 23558749 |
| 199 | uc.38 | BC | downregulated | ✘ | ✘ | ✔ | 29312798 |
| 200 | uc.57 | BC | downregulated | ✔ | ✘ | ✘ | 29179465 |
| 201 | uc.63 | BC | upregulated | ✘ | ✘ | ✘ | 27447964 |
| 202 | UCA1 | BC | upregulated | ✔ | ✘ | ✘ | 29408336 |
|  | UCA1 | BC | upregulated | ✘ | ✘ | ✘ | 29669595 |
|  | UCA1 | BC | upregulated | ✔ | ✘ | ✘ | 27629141 |
|  | UCA1 | BC | upregulated | ✘ | ✔ | ✘ | 28248879 |
|  | UCA1 | BC | upregulated | ✔ | ✘ | ✘ | 27831634 |
|  | UCA1 | BC | differential expression | ✘ | ✘ | ✔ | 27697109 |
|  | UCA1 | BC | differential expression | ✘ | ✘ | ✘ | 27424981 |
|  | UCA1 | BC | upregulated | ✔ | ✘ | ✘ | 27765938 |
|  | UCA1 | BC | upregulated | ✘ | ✘ | ✘ | 26464647 |
|  | UCA1 | BC | upregulated | ✘ | ✘ | ✘ | 24457952 |
|  | UCA1 | BC | upregulated | ✔ | ✘ | ✘ | 17416635 |
|  | UCA1 | BC | upregulated | ✘ | ✘ | ✘ | 26439035 |
|  | UCA1 | BC | upregulated | ✔ | ✘ | ✔ | 27977766 |
|  | UCA1 | BC | upregulated | ✘ | ✘ | ✘ | 26550436 |
|  | UCA1 | BC | upregulated | ✘ | ✘ | ✔ | 29774079 |
| 203 | X15675 | BC | upregulated | ✔ | ✘ | ✘ | 28003470 |
| 204 | XIST | BC | downregulated | ✘ | ✘ | ✘ | 27248326 |
|  | XIST | BC | downregulated | ✔ | ✘ | ✘ | 24141629 |
|  | XIST | BC | differential expression | ✘ | ✘ | ✘ | 17545591 |
|  | XIST | BC | upregulated | ✘ | ✘ | ✘ | 29550489 |
|  | XIST | BC | upregulated | ✘ | ✘ | ✘ | 19440381 |
| 205 | XR_250621.1 | TNBC | upregulated | ✘ | ✘ | ✘ | 26078338 |
| 206 | Yiya | BC | upregulated | ✘ | ✘ | ✔ | 29967256 |
| 207 | Z38 | BC | upregulated | ✘ | ✘ | ✔ | 28247935 |
|  | Z38 | BC | upregulated | ✘ | ✘ | ✘ | 27053956 |
| 208 | ZFAS1 | BC | downregulated | ✘ | ✘ | ✔ | 29532866 |
|  | ZFAS1 | BC | downregulated | ✘ | ✘ | ✘ | 27871336 |
|  | ZFAS1 | BC | downregulated | ✘ | ✘ | ✘ | 21460236 |
| 209 | ZFHX4-AS1 | BC | upregulated | ✘ | ✔ | ✔ | [30546116](http://www.ncbi.nlm.nih.gov/pubmed/?term=30546116) |

BC: breast cancer, TNBC: triple-negative breast cancer. The studies related to the specified lncRNA Cyclin-Dependent Kinase Inhibitor 2B Antisense RNA 1 (i.e. ANRIL) in the present work have been coloured. Data source: lnc2cancer database.

**Table S2 Overlapping transcripts for rs2383207 variant according to NCBI and Varsome.com**

| **CDKN2B-AS1 Transcript** | **Location** | **Splice distance** |
| --- | --- | --- |
| NR_047543.1 | intron 6 of 8 position 2161 of 4844 | 2161 |
| NR_003529.3 | intron 16 of 18 position 2161 of 4844 | 2161 |
| NR_047532.1 | intron 11 of 13 position 2161 of 4844 | 2161 |
| NR_047534.1 | intron 6 of 8 position 2161 of 4844 | 2161 |
| NR_047535.1 | intron 5 of 7 position 3565 of 6248 | -2684 |
| NR_047536.1 | intron 4 of 6 position 3565 of 6248 | -2684 |
| NR_047537.1 | intron 4 of 5 position 59573 of 63812 | -4240 |
| NR_047538.1 | intron 3 of 4 position 66732 of 70971 | -4240 |
| NR_120536.1 | intron 3 of 3 position 66732 of 71275 | -4544 |

**Table S3 Role of CDKN2B-AS1 in cancer as a diagnostic and prognostic biomarker and therapeutic target (a review of the previous literature)**

| **Disease name** | **Dysfunction type** | **Description** | **Reference** |
| --- | --- | --- | --- |
| Bladder cancer | Locus | CDKN2B-AS1 possibly serves as an oncogene in bladder cancer and regulates bladder cancer cell proliferation and apoptosis through the intrinsic apoptosis pathway. | [26449463](http://www.ncbi.nlm.nih.gov/pubmed/26449463) |
| Breast cancer | Expression | The expression of CDKN2B-AS1 was mainly co-clustered with p14/ARF both in physiologic (various normal human tissues) and in pathologic conditions (human breast tumors). | [17440112](http://www.ncbi.nlm.nih.gov/pubmed/17440112) |
|  | Expression | It can be used as a potential cancer biomarker. | [26409453](http://www.ncbi.nlm.nih.gov/pubmed/26409453) |
|  | Expression | The expression level of ANRIL was upregulated in TNBC tumor tissue and cell lines compared to noncancerous tissue and non-TNBC cells. Besides, the upregulated ANRIL expression was closely correlated to poor prognosis. In vitro, loss-of-function experiments showed that ANRIL knockdown interfered by interference oligonucleotide could markedly suppress TNBC cell proliferation and enhance apoptosis. In vivo, ANRIL knockdown inhibited tumor growth. | **28961506** |
|  | Expression | ANRIL, HIF1A-AS2, and UCA1 expression were significantly increased in plasma of patients with TNBC, suggesting their use as TNBC-specific diagnostic biomarkers. | **28248879** |
|  | Locus | A locus associated with breast cancer. | [20453838](http://www.ncbi.nlm.nih.gov/pubmed/20453838)  [20956613](http://www.ncbi.nlm.nih.gov/pubmed/20956613)  [27102007](http://www.ncbi.nlm.nih.gov/pubmed/27102007) |
|  | Regulation | Expression analysis of ANRIL, EZH2, SUZ12, EED, JARID2, CBX7, BMI1, p16, p15, and p14/ARF genes was evaluated in a large cohort of invasive breast carcinomas. | **27102007** |
|  | Mutation | Four haplotypes (rs1333045, rs4977574, rs1333048, and rs10757278) were associated with breast cancer risk. | **28580310** |
|  | Mutation | Additional GWAS identified CDKN2B-AS1 as a risk locus (rs3217992, A>G; rs1063192, C>T) for several cancers, including breast cancer, nasopharyngeal carcinoma, basal cell carcinoma, & glioma. | [20956613](http://www.ncbi.nlm.nih.gov/pubmed/20956613) |
| Cervical cancer | Regulation | Long Noncoding RNA CDKN2B-AS1 Regulates Proliferation of Non-small Cell Lung Cancer and Cervical Cancer Cells. | [26408699](http://www.ncbi.nlm.nih.gov/pubmed/26408699) |
|  | Expression | lncRNA ANRIL was highly expressed in CC and participated in the regulation of migration, proliferation, and invasion of CC cells. Inhibition of ANRIL expression led to decreased expressions of Cyclin D1, CDK4, CDK6, N-cadherin, and Vimentin, along with attenuated cell proliferation, migration, and invasion of CC cells. | **30198868** |
|  | Expression | High ANRIL expression is associated with Cervical Cancer Development. Long Noncoding RNA ANRIL Promotes Cervical Cancer Development by Acting as a Sponge of miR-186. | **28550682** |
|  | Expression | The expression of lncRNA ANRIL was significantly increased both in cervical cancer tissues and cell lines. Patients with high ANRIL expression had advanced FIGO stage, lymph node metastasis, and poor overall survival than those with low ANRIL expression. Loss-of-function experiments showed that decreased expression of ANRIL inhibited cell proliferation, migration, and invasion of cervical cancer. | **27899255** |
| Colorectal cancer | Regulation | CDKN2B-AS1 is associated with the survival rate of patients with colorectal cancer and affects cell migration and invasion *in vitro*. | [27314206](http://www.ncbi.nlm.nih.gov/pubmed/27314206) |
|  | Regulation | CDKN2B-AS1 positively regulates the proliferation of HCT116 cells in two- and three-dimensional culture in a p15/p16-pRB pathway-independent manner. | [26708220](http://www.ncbi.nlm.nih.gov/pubmed/26708220) |
|  | Expression | It was upregulated in tumor tissue samples from patients with CRC and CRC cell lines. ANRIL overexpression significantly promoted cell chemoresistance by regulating ATP-binding cassette subfamily C member 1 through binding Let-7a. ANRIL knockdown significantly inhibited CRC cell proliferation, improved the sensitivity of chemotherapy, and promoted apoptosis. | 30279206 |
|  | Expression | Downregulation of long non-coding RNA CDKN2B-AS1 suppresses lymphangiogenesis and lymphatic metastasis in colorectal cancer. | [27286457](http://www.ncbi.nlm.nih.gov/pubmed/27286457) |
|  | Expression | The CRC tissues were revealed to express higher levels of ANRIL lncRNA compared with the adjacent nonneoplastic tissues. High ANRIL expression was significantly associated with a reduced survival rate. | **27314206** |
| Esophageal cancer | Regulation | The chemotherapeutic drug β-elemene suppressed the proliferation of esophageal carcinoma ECA-109 cells by regulating the inhibition of hTERT expression by lncRNA CDKN2B-AS1. | [25646744](http://www.ncbi.nlm.nih.gov/pubmed/25646744) |
|  | Mutation | ANRIL rs2151280 T/C was not associated with the risk of ESCC. | **25874495** |
|  | Expression | a significant role of ANRIL in the occurrence and development of esophageal squamous cell carcinoma through TGFbeta1 signaling pathways. | **24747824** |
|  | Expression | High ANRIL expression is associated with esophageal squamous cell carcinoma. | **30610814** |
| Gallbladder cancer | Regulation | CDKN2B-AS1 can improve the proliferation of gallbladder cells and inhibit apoptosis. | [26812694](http://www.ncbi.nlm.nih.gov/pubmed/26812694) |
| Gastric cancer | Expression | Long non-coding RNA CDKN2B-AS1 indicates a poor prognosis of gastric cancer and promotes tumor growth by epigenetically silencing of miR-99a/miR-449a. | [24810364](http://www.ncbi.nlm.nih.gov/pubmed/24810364) |
|  | Regulation | Knockdown of lncRNA CDKN2B-AS1 in gastric cancer cells inhibits the development of MDR, suggesting an efficient target for reversing MDR in gastric cancer therapy. | [27121324](http://www.ncbi.nlm.nih.gov/pubmed/27121324) |
|  | Regulation | CDKN2B-AS1 knockdown blocks the effects of TET2 on gastric cancer cell proliferation and colony formation. | [27027260](http://www.ncbi.nlm.nih.gov/pubmed/27027260) |
|  | Expression | Knockdown of ANRIL suppressed cell viability, migration, and invasion, and increased apoptosis through upregulating miR-99a. ANRIL silence downregulated BMI1 via upregulating miR-99a. BMI1 silence downregulated Bcl-2 and key kinases in the Notch and mTOR pathways and upregulated p16 and cleaved caspases. We verified the tumor-suppressive effects of ANRIL knockdown in gastric cancer cells via crosstalk with miR-99a | **30156609** |
|  | Expression | The knockdown of lncRNA ANRIL in gastric cancer cells inhibits the development of MDR. | **27121324** |
|  | Regulation | It plays an important role in gastric carcinogenesis | [25636450](http://www.ncbi.nlm.nih.gov/pubmed/25636450) |
|  | Regulation | ANRIL expression in gastric cancer cell lines and tumor tissues was negatively associated with the level of PLZF and with ANRIL recruited polycomb repressive complex 2, which then drove PLZF silencing by collaborating between H3K27me3 and DNA methylation. | **30431129** |
| Hepatocellular carcinoma | Expression | ANRIL can suppress HCC development by regulating miR-384 and STAT3. ANRIL expression was upregulated in HCC cells, including SMCC7721, HepG2, MHCC-97H, SNU449, and HUH-7 cells, in comparison to the normal human liver cells LO2. Knockdown of ANRIL suppressed HCC cell proliferation and induced cell cycle arrest and apoptosis. HCC cell migration and invasion capacity were inhibited by inhibition of ANRIL. | 31679275 |
|  | Expression | ANRIL knockdown suppressed proliferation, migration, invasion, and promoted apoptosis in HepG2 cells by down-regulating miR-191 and inactivating NF-κB and Wnt/β-catenin signaling pathways. | 30249208 |
|  | Expression | lncRNA CDKN2BAS promotes HCC metastasis by regulating the miR-153-5p/ARHGAP18 signaling. | **30510148** |
|  | Expression | Long non-coding RNAs (lncRNAs) CDKN2B-AS1 promoted nucleosome assembly protein 1 like 1 (NAP1L1) expression by sponging let-7c-5p in hepatocellular carcinoma (HCC) cells. | **30165194** |
|  | Expression | lncRNA ANRIL expression in HCC tissues was significantly higher. High lncRNA ANRIL expression was an independent predictor of poor prognosis. *In vitro* assays revealed that the decreased expression of lncRNA ANRIL could suppress cell proliferation, migration, and invasion of HCC cells. | **26045820** |
|  | Expression | ANRIL knockdown impeded tumor growth. ANRIL was upregulated in HCC tissues. Knockdown of ANRIL suppressed cell proliferation, metastasis, and invasion via regulating miR-122-5p expression in HCC. | 29127494 |
|  | Expression | knockdown of ANRIL expression could impair cell proliferation and invasion and induce cell apoptosis both in vitro and in vivo. The authors also found that ANRIL could epigenetically repress KLF2 transcription in HCC cells by binding with PRC2 and recruiting it to the KLF2 promoter region. | **27391317** |
| Gall bladder | Expression | High ANRIL expression is associated with gallbladder cancer. ANRIL can improve the proliferation of gallbladder cells and inhibit apoptosis. | **26812694** |
| Pancreatic cancer | Expression | Over-expression of lncRNA ANRIL can promote EMT of PC cells by activating the ATM-E2F1 signaling pathway. In rat models, ANRIL interference promoted the expressions of INK4B, INK4A, ARF, and E-cadherin, while reduced N-cadherin and Vimentin expression. | **28344092** |
| Head and neck squamous cell carcinoma | Expression | ANRIL promotes G1 phase progression by repressing p15 and p16, and thus promotes FaDu and CAL27 cell proliferation. | **31366490** |
| Laryngeal Squamous Cell Cancer | Expression | The expression of CDKN2B-AS1 was significantly higher in LSCC tissues than in adjacent normal tissues. Higher CDKN2B-AS1 was closely associated with lymph node metastasis and advanced clinical stage. CDKN2B-AS1 knockdown by siRNA significantly inhibited the proliferation, induced cell apoptosis, and suppressed migration and invasion in LSCC cells. | 31754305 |
| Nasopharyngeal carcinoma | Expression | The upregulation of ANRIL correlates with nasopharyngeal carcinoma progression. | 29463902 |
|  | Expression | The expression of lncRNA ANRIL was upregulated in nasopharyngeal carcinoma (NPC) tissues and cells. Moreover, knockdown of ANRIL repressed proliferation, promoted apoptosis, and improved radiosensitivity in NPC via functioning as a miR-125a sponge | **28402230** |
|  | Expression | knockdown of ANRIL represses tumorigenicity and enhances DDP-induced cytotoxicity via regulating microRNA let-7a in NPC cells | **28117929** |
|  | Expression | ANRIL could promote nasopharyngeal carcinoma progression via increasing cell proliferation, reprogramming cell glucose metabolism, and inducing side-population stem-like cancer cells. ANRIL can also reprogram glucose metabolism via increasing glucose uptake for glycolysis, which was regulated by the mTOR signal pathway to affect the expression of essential genes in glycolysis. | **27557514** |
| Lung cancer | Expression | It may be potential clinical biomarkers to predict lung cancer risk and platinum-based chemotherapy response. | [26729200](http://www.ncbi.nlm.nih.gov/pubmed/26729200) |
|  | Regulation | CDKN2B-AS1 is a lncRNA responsible for anti-tumorigenesis caused by PLD inhibition and combined incorporation of CDKN2B-AS1 into PLD inhibition. | [25964559](http://www.ncbi.nlm.nih.gov/pubmed/25964559) |
|  | Regulation | Increased expression of the long non-coding RNA CDKN2B-AS1 promotes lung cancer cell metastasis and correlates with poor prognosis. | [25889788](http://www.ncbi.nlm.nih.gov/pubmed/25889788) |
|  | Expression | Lung adenocarcinoma (LAD) patients' paclitaxel treatment and made ANRIL be a new target for paclitaxel-based chemotherapy in LAD | **28402932** |
| Non-small cell lung cancer | Regulation | Long non-coding RNA CDKN2B-AS1 could be transactivated by cMyc and promote tumor progression of non-small-cell lung cancer. | [27307748](http://www.ncbi.nlm.nih.gov/pubmed/27307748) |
|  | Expression | Circulating SPRY4-IT1, CDKN2B-AS1, and NEAT, might serve as a predictor for the early warning of non-small-cell lung cancer. | [26453113](http://www.ncbi.nlm.nih.gov/pubmed/26453113) |
|  | Regulation | Long Noncoding RNA CDKN2B-AS1 Regulates Proliferation of Non-small Cell Lung Cancer and Cervical Cancer Cells. | [26408699](http://www.ncbi.nlm.nih.gov/pubmed/26408699) |
|  | Regulation | Long non-coding RNA CDKN2B-AS1 promotes non-small cell lung cancer cell proliferation and inhibits apoptosis by silencing KLF2 and P21 expression. | [25504755](http://www.ncbi.nlm.nih.gov/pubmed/25504755) |
| Thyroid cancer | Regulation | Long non-coding RNA CDKN2B-AS1 promotes the invasion and metastasis of thyroid cancer cells through TGF-β/Smad signaling pathway. | [27507052](http://www.ncbi.nlm.nih.gov/pubmed/27507052) |
| Melanoma | Expression | High expression of CDKN2B-AS1 has been found in certain cancer tissues such as melanoma and prostate cancers. | [24757675](http://www.ncbi.nlm.nih.gov/pubmed/24757675) |
|  | Mutation | Complex rearrangements (fusion transcripts) involving MTAP and ANRIL in two of the seven primary melanoma tumors with focal deletion at the locus. | **26909863** |
|  | Regulation | This study found differential expression of ANRIL exons, which indicates that multiple isoforms exist in melanoma cells. Multiple isoforms exist in melanoma cells. In addition to linear isoforms, we identified circular forms of *ANRIL* (*circANRIL*). | **28653984** |
|  | N/A | Long ncRNA antisense non-coding RNA in the INK4 locus (CDKN2B-AS1) has been associated with hereditary cutaneous malignant melanoma, prostate cancer, and tumors of the neural system. | [24624135](http://www.ncbi.nlm.nih.gov/pubmed/24624135) |
|  | Expression | In a suspicious INK4b-ARF-INK4a gene cluster at chromosome 9p21 in uveal melanoma cells, aberrant INK4a and INK4b defects were simultaneously endogenously auto-corrected after targeting the suppression of abnormal ANRIL lncRNA. | **27461581** |
| Glioma | Locus | Furthermore, genome-wide association studies have identified the CDKN2B-AS1 gene as a risk locus for coronary disease, intracranial aneurism, type 2 diabetes, and several cancers, including glioma. | [24624135](http://www.ncbi.nlm.nih.gov/pubmed/24624135) |
| Ovarian cancer | Regulation | CDKN2B-AS1 promotes EOC (epithelial ovarian cancer) progression and is a potential prognostic biomarker. Down-regulation of P15INK4B and up-regulation of Bcl-2 by ANRIL may partially explain ANRIL-induced EOC cell proliferation. | [27095571](http://www.ncbi.nlm.nih.gov/pubmed/27095571) |
|  | Regulation | LncRNA CDKN2B-AS1 plays an important role in SOC invasion/metastasis and could represent a novel biomarker for predicting poor survival as well as a promising therapeutic target. | [25845387](http://www.ncbi.nlm.nih.gov/pubmed/25845387) |
| Prostate cancer | Expression | Recent studies have linked their misexpression to diverse cancers. | [23660942](http://www.ncbi.nlm.nih.gov/pubmed/23660942) |
|  | Regulation | Gene silencing of INK4b-ARF-INK4a and p15/CDKN2B by recruitment of PRC1 and PRC2. | [22996375](http://www.ncbi.nlm.nih.gov/pubmed/22996375) |
|  | Regulation | CDKN2B-AS1 is an antisense lncRNA elevated in PCa that overlaps this locus, interacting directly with polycomb repressive complex 1 and histone H3K27 methylation to repress CDKN2A-CDKN2B expression | [24146262](http://www.ncbi.nlm.nih.gov/pubmed/24146262) |
|  | Interaction | Here we report that chromobox 7 (CBX7) within the polycomb repressive complex 1 binds to CDKN2B-AS1, and both CBX7 and CDKN2B-AS1 are found at elevated levels in prostate cancer tissues. | [20541999](http://www.ncbi.nlm.nih.gov/pubmed/20541999) |
|  | N/A | Long ncRNA antisense non-coding RNA in the INK4 locus (CDKN2B-AS1) has been associated with hereditary cutaneous malignant melanoma, prostate cancer, and tumors of the neural system. | [24624135](http://www.ncbi.nlm.nih.gov/pubmed/24624135) |
|  | Expression | Overexpression of lncRNA ANRIL promoted the proliferation and migration of prostate cancer cells via regulating let-7a/TGF-beta1/Smad signaling pathway. | **29278879** |
|  | Expression | CDKN2B-AS1 is upregulated in prostate cancer and is required for the repression of the tumor suppressors INK4a/p16 and INK4b/p15. | [24721780](http://www.ncbi.nlm.nih.gov/pubmed/24721780) |
|  | Mutation | Antisense non-coding RNA in the INK4 locus (rs1333045, rs4977574, rs1333048, and rs10757278) possibly participates in the pathogenesis of Prostate Cancer and Benign Prostatic Hyperplasia in an Iranian Population | **28621612** |
| Renal cell carcinoma | Expression | ANRIL was highly expressed in RCC tissues and RCC cell lines. ANRIL significantly promoted cell proliferation, migration, invasion, and EMT but inhibited cell apoptosis. Additionally, the expression levels of β-catenin, Ki-67, glycogen synthase kinase 3β (GSK-3β), phosphorylated GSK-3β, T-cell transcription factor 4 (TCF-4), and leukemia enhancer factor 1 (LEF-1) were all markedly upregulated by ANRIL. The effect of ARNIL silencing was opposite to that of ANRIL overexpression. | **28251886** |
| Osteosarcoma | Expression | Our study demonstrates that ANRIL knockdown sensitizes osteosarcoma cells to cisplatin-induced cytotoxicity. | **30777616** |
| Multiple myeloma | Expression | Polymorphism in ANRIL is associated with relapse in multiple myeloma. ANRIL may be involved in melphalan-mediated apoptosis via down-regulating p14ARF and subsequent p53, and that the rs2151280 polymorphism may be a potential prognostic biomarker for relapse in melphalan-treated MM patients. | **28150872** |
| Leukemia | Expression | Another lncRNA associated with human cancers is CDKN2B-AS1, a long, antisense transcript found in the INK4a/Arf locus. CDKN2B-AS1 is overexpressed in human leukemias and prostate cancers, and its expression leads to epigenetic silencing of the nearby tumor suppressor p15 (Yu et al., 2008; Yap et al., 2010). | [23473599](http://www.ncbi.nlm.nih.gov/pubmed/23473599) |
|  | Expression | Long Noncoding RNA ANRIL Supports Proliferation of Adult T-Cell Leukemia Cells Through Cooperation with EZH2. Results implicate that the lncRNA ANRIL, by cooperating with EZH2, supports the proliferation of HTLV-1-infected cells, which is thought to be critical for oncogenesis. | **30258009** |
| Medulloblastoma | Mutation | CDKN2BAS rs2157719 T>C genetic polymorphism was significantly associated with an increased medulloblastoma risk. | **29314442** |
|  | Expression | ANRIL acted as a sponge of miR-323, and its silence functioned through upregulating miR-323. BRI3 and CDK6 were target genes of miR-323, and the effect of BRI3 on DAOY cells was the same as ANRIL. Moreover, ANRIL suppression could reduce phosphorylated levels of p38 MAPK, ERK, and AKT, and inhibit the Wnt signaling pathway through positively regulating BRI3. | **28513871** |
| Glioma | Expression | ANRIL was upregulated in glioma, and its inhibition could repress cell proliferation, migration, and invasion but inhibit cell apoptosis through miR-34a-mediated downregulation of Sirt1, involving the inactivation of the PI3K/AKT and mTOR pathways. | **29057547** |
|  | Mutation | Results found an association between rs1412829 and rs4977756 (9p21.3, CDKN2B-AS1) and global DNA methylation pattern in glioma, for which a trend was also seen in the TCGA glioblastoma dataset. | **27780202** |
| Retinoblastoma | Expression | The silence of lncRNA ANRIL Represses Cell Growth and Promotes Apoptosis in Retinoblastoma Cells Through Regulating miR-99a and c-Myc | **31184221** |
|  | Expression | The Silencing of Long Non-Coding RNA ANRIL Suppresses invasion and promotes Apoptosis of Retinoblastoma Cells Through the ATM-E2F1 Signaling Pathway. ANRIL negatively regulates the ATM-E2F1 signaling pathway. The down-regulation of ANRIL or up-regulation of ATM led to an increase in the expressions of ATM, E2F1, INK4b, INK4a, ARF, p53, and pRB. | **30355646** |
|  | expression | Overexpression of ANRIL promoted viability, migration, and invasion of retinoblastoma cells by activating MEK/ERK and Wnt/β-catenin pathways, as well as down-regulating miR-24 and then upregulating c-Myc. | **30703428** |
| Cancer | Expression | Downregulated in cells were incubated in the presence of BLM for 24 h or irradiated. | [22487937](http://www.ncbi.nlm.nih.gov/pubmed/22487937) |
|  | Interaction | RNA immunoprecipitation demonstrates that CDKN2B-AS1 binds to SUZ12 in vivo. Collectively, these results suggest a model in which CDKN2B-AS1 binds to and recruits PRC2 to repress the expression of p15(INK4B) locus. | [21151178](http://www.ncbi.nlm.nih.gov/pubmed/21151178) |
|  | Regulation | The large non-coding RNA CDKN2B-AS1, associated with atherosclerosis, periodontitis, and several forms of cancer, regulates ADIPOR1, VAMP3, and C11ORF10. | [23813974](http://www.ncbi.nlm.nih.gov/pubmed/23813974) |
|  | Regulation | It has also been shown that the tumor suppressor gene p15 is silenced by its natural antisense RNA, a lncRNA CDKN2B-AS1. | [24829860](http://www.ncbi.nlm.nih.gov/pubmed/24829860) |
|  | Expression | CDKN2B-AS1 is a recently discovered long non-coding RNA encoded in the chromosome 9p21 region. This locus is a hotspot for disease-associated polymorphisms, and it has been consistently associated with cardiovascular disease, and more recently, with several cancers, diabetes, glaucoma, endometriosis, among other conditions. | [23104877](http://www.ncbi.nlm.nih.gov/pubmed/23104877) |
|  | Regulation | Molecular interplay between non-coding RNA and H3K27me as mediated by the conserved chromodomain. | **20541999** |
|  | Regulation | lncRNA-mediated regulation of the INK4b-ARF-INK4a gene is not restricted to ANRIL; both polycomb repressive complex-1 (PRC1) and -2 (PRC2) interact with ANRIL to form heterochromatin surrounding the INK4b-ARF-INK4a locus | **21828241** |
|  | Regulation | An elevated level of ANRIL suppresses the expression of INK4a, INK4b, and ARF at the late-stage of the DNA damage response. | **23416462** |
|  | Regulation | CDKN2B-AS1, GAS5, and lincRNA-p23 are involved in escaping growth suppression by regulating tumor suppressor genes (CDKN2B-AS1) or apoptosis regulators. | [24667321](http://www.ncbi.nlm.nih.gov/pubmed/24667321) |
